# Causal association between lipid-lowering drugs and female reproductive endocrine diseases: a drug-targeted Mendelian randomization study

**DOI:** 10.3389/fendo.2023.1295412

**Published:** 2023-11-10

**Authors:** Jing Zhao, Runfang Wang, Liyun Song, Hua Han, Pei Wang, Yuan Zhao, Yunxia Zhang, Hongzhen Zhang

**Affiliations:** ^1^ Department of Gynecology, Hebei General Hospital, Shijiazhuang, China; ^2^ Department of Obstetrics, Hebei General Hospital, Shijiazhuang, China; ^3^ Department of Clinical Laboratories, Kunhua Affiliated Hospital, Kunming University of Science and Technology, Kunming, China; ^4^ Department of Obstetrics and Gynecology, The First Hospital of Hebei Medical University, Shijiazhuang, China

**Keywords:** lipid-lowering drugs, female reproductive endocrine diseases, eQTL, drug-target, Mendelian randomization

## Abstract

**Purpose:**

The relationship between dyslipidemia and female reproductive endocrine diseases has been increasingly studied. The use of lipid-lowering drugs in treating various related diseases, including coronary heart disease, may affect female reproductive endocrine diseases. Therefore, our study aims to investigate the effects of lipid-lowering drugs on female reproductive endocrine diseases and provide a basis for the appropriate selection of drugs.

**Methods:**

In this study, we focused on three drug targets of statins, namely HMG-CoA reductase (HMGCR) inhibitors, proprotein convertase kexin 9 (PCSK9) inhibitors, and Niemann–Pick C1-Like 1 (NPC1L1) inhibitors. To identify potential inhibitors for these targets, we collected single nucleotide polymorphisms (SNPs) associated with HMGCR, PCSK9, and NPC1L1 from published genome-wide association study statistics. Subsequently, we conducted a drug target Mendelian randomization (MR) analysis to investigate the effects of these inhibitors on reproductive endocrine diseases mediated by low-density lipoprotein cholesterol (LDL-C) levels. Alongside coronary heart disease as a positive control, our main outcomes of interest included the risk of polycystic ovary syndrome (PCOS), premature ovarian insufficiency (POI), premenstrual syndrome (PMS), abnormal uterine bleeding (including menorrhagia and oligomenorrhea), and infertility.

**Results:**

PCSK9 inhibitors significantly increased the risk of infertility in patients (OR [95%CI] = 1.14 [1.06, 1.23], p<0.05). In contrast, HMGCR inhibitors significantly reduced the risk of menorrhagia in female patients (OR [95%CI] = 0.85 [0.75, 0.97], p<0.05), but had no statistical impact on patients with oligomenorrhea.

**Conclusion:**

The findings suggest that PCSK9 inhibitors may significantly increase the risk of infertility in patients. On the other hand, HMGCR inhibitors could potentially offer protection against menorrhagia in women. However, no effects of lipid-lowering drugs have been observed on other reproductive endocrine disorders, such as PCOS, POF, PMS and oligomenorrhea.

## 1 Introduction

Reproductive endocrine diseases are a prevalent type of gynecological conditions that typically result from abnormalities in the hypothalamic-pituitary-ovarian axis function or abnormal effects on target organs, such as polycystic ovary syndrome (PCOS), premature ovarian failure (POF), premenstrual syndrome (PMS), abnormal uterine bleeding, infertility, and other related diseases. It has been noted that cholesterol can generate estradiol, progesterone, and testosterone via the Δ^4^ and Δ^5^ pathway (1). These sex hormones play a significant role in the onset and progression of reproductive endocrine diseases. Recent studies have increasingly indicated a close association between abnormal blood lipid levels and female reproductive endocrine diseases. Animal studies have demonstrated that quercetin and rosuvastatin play a role in restoring ovarian function, leading to significant and dose-dependent increases in the number of primordial follicles, serum anti-Müllerian hormone levels, and ovarian tissue glutathione levels. Importantly, these effects were observed without compromising its anti-tumor properties (2). In a study by Cerin et al., hormonal parameters, glucose tolerance, mineralocorticoids, cholesterol, triglycerides, apolipoprotein (a), magnesium, and calcium were compared. The results indicated that cholesterol levels during the follicular phase were significantly higher in women with PMS (3). Sharami et al.’s study suggested a positive correlation between low-density lipoprotein cholesterol (LDL-C) levels and abnormal uterine bleeding caused by uterine fibroids (4). Furthermore, a recent single-multivariable Mendelian randomization (MR) study revealed a positive correlation between elevated LDL-C levels and the risk of infertility (5). In summary, we can’t help but wonder whether lipid-lowering drugs have an impact on female reproductive endocrine diseases.

3-Hydroxy-9-methylglutaryl-coenzyme A reductase (HMGCR) inhibitors, specifically statins like simvastatin and rosuvastatin, are widely used lipid-lowering drugs that have multiple effects. These drugs have been thoroughly confirmed to be safe, cost-effective, and have pleiotropic effects (6). In a meta-analysis conducted by Raval et al, which included 4 randomized controlled trials (RCTs) involving 244 women with PCOS, it was found that statins, either alone or in combination with oral contraceptives (OCPs), effectively reduce testosterone levels and improve total cholesterol, LDL, and triglyceride levels (6). Another study by Xu et al. examined 287 PCOS patients and 187 non-PCOS controls which revealed that variations in the HMGCR gene may impact the compositional characteristics of PCOS, including insulin resistance, triglyceride levels, and free testosterone levels (7). In menopausal women, low estrogen levels can lead to dyslipidemia, increasing the risk of premature cardiovascular disease. Research has demonstrated that estradiol (E2) can increase HMGCR and low-density lipoprotein receptor (LDLR) expression and lipid secretion in Human hepatoblastoma (HepG2) (8). Furthermore, simvastatin has been shown to inhibit the proliferation of human endometrial stromal (HES) cells and reduce the number and size of endometrial implants in a nude mouse model of endometriosis, suggesting the potential benefits of HMGCR inhibitors in treating infertility (9).

Proprotein convertase subtilis kexin 9 (PCSK9) is a serine protease that plays a crucial role in regulating LDL-C metabolism (10). It has been implicated in the development of dyslipidemia and atherosclerosis by hindering the recycling of LDLR back to the cell surface, resulting in elevated levels of LDL-C. Certain missense mutations in the PCSK9 gene can cause dominant hypercholesterolemia in patients by increasing PCSK9 activity. In these hypercholesterolemic patients, plasma levels of LDL-C were approximately 50% higher than in patients with either mutation alone (11). Studies have shown that the D374Y mutant of PCSK9 (12) has 10 times higher affinity to the LDLR compared to the wild type (13). On the other hand, certain nonsense, missense, or in-frame deletion mutations found in the PCSK9 gene can lead to hypocholesterolemia by increasing LDL-C clearance. A 15-year prospective study demonstrated that nonsense heterozygous mutations in PCSK9 not only reduced LDL-C levels by 28% but also decreased the incidence of coronary heart disease by 88%. In the case of mutations in the R46L allele, the incidence of coronary heart disease is reduced by 50%, while LDL-C levels are reduced by an average of 15% (14). A study conducted by Luana B Xavier et al. discovered a correlation between PCSK9 and lipid and androgen metabolism in Brazilian women with PCOS (15). In addition to its crucial role in maintaining lipid homeostasis, PCSK9 is also associated with various signaling pathways, such as enhancing viral activity (16), participating in cell apoptosis (17), and contributing to the anti-tumor immune response (18).

Niemann-Pick C1-Like 1 (NPC1L1) is a protein that plays a crucial role in regulating circulating levels of LDL-C (19). According to Altmann et al. (20), NPC1L1 plays a crucial role in intestinal cholesterol absorption. NPC1L1 is a transmembrane protein found in multiple cells, particularly in the apical membrane of enterocytes and the renal tubular membrane of hepatocytes. It functions as a sterol transporter and is responsible for mediating intestinal cholesterol absorption and maintaining hepatobiliary cholesterol excretion (21). Studies have shown that NPC1L1 inhibitors effectively lower both human serum and bile cholesterol levels (22). Therefore, inhibiting the function or gene expression of NPC1L1 in intestinal epithelial cells holds promise as a potential strategy for regulating intestinal cholesterol absorption and treating hypercholesterolemia (23).

Presently, the FDA has approved PCSK9 inhibitors (evolocumab and alirocumab) and NPC1L1 inhibitors (ezetimibe) as effective lipid-lowering drugs (24, 25). However, there is currently no research available on lipid-lowering drugs’ correlation with female reproductive endocrine diseases. The potential use of lipid-lowering drugs in treating female reproductive endocrine diseases is evident. Presently, research and guidelines only suggest the use of these drugs for individuals with dyslipidemia, without specifying the specific type of lipid-lowering drug to be used or its impact on female adolescents. The effects of these drugs on reproductive endocrinology remain inconclusive.

Mendelian randomization (MR) utilizes instrumental variables (IV) to investigate the causal association between exposure and outcome (26). It provides evidence that falls between RCTs and observational studies (27). According to Mendel’s laws of inheritance, alleles are randomly passed from parents to offspring, resulting in offspring genotypes that are largely independent of confounding factors. Consequently, MR analysis can better circumvent reverse causality and the potential influence of confounding factors compared to traditional observational studies (27). Additionally, the operational environment and complexity of MR analysis are significantly lower than those of RCTs. Drug target MR analysis is a method that simulates the genetic variation of pharmacological inhibition of drug-gene targets. The regression estimates obtained from this analysis reflect the long-term impact of drug use, which strengthens the causal inference regarding the potential impact of these drug-gene targets on female reproductive endocrine diseases. The level of evidence provided by MR analysis is considered to be second only to randomized controlled studies (19, 25, 27, 28). In a case-control study, the odds ratio (OR) can be calculated by dividing the ratio of the number of exposed persons to the number of unexposed persons in the case group by the ratio of the number of exposed persons to the number of unexposed persons in the control group. This ratio can be interpreted as a risk ratio, rate ratio, odds ratio, or prevalence ratio. The odds ratio, often referred to as the OR value, is also applicable to MR analysis (29). In this study, we collected summary-level statistical data from recently published genome-wide association studies (GWAS) and performed drug-targeted MR analysis to investigate the causal relationship between lipid-lowering drugs (HMGCR, PCSK9, and NPC1L1 inhibitors) and female reproductive endocrine diseases. These diseases include PCOS, POF, PMS, abnormal uterine bleeding, and infertility. We also considered coronary heart disease (CHD) as a positive control.

## 2 Methods

### 2.1 Selection of genetic instrumental variables for three lipid-lowering drugs

**Figure 1:**
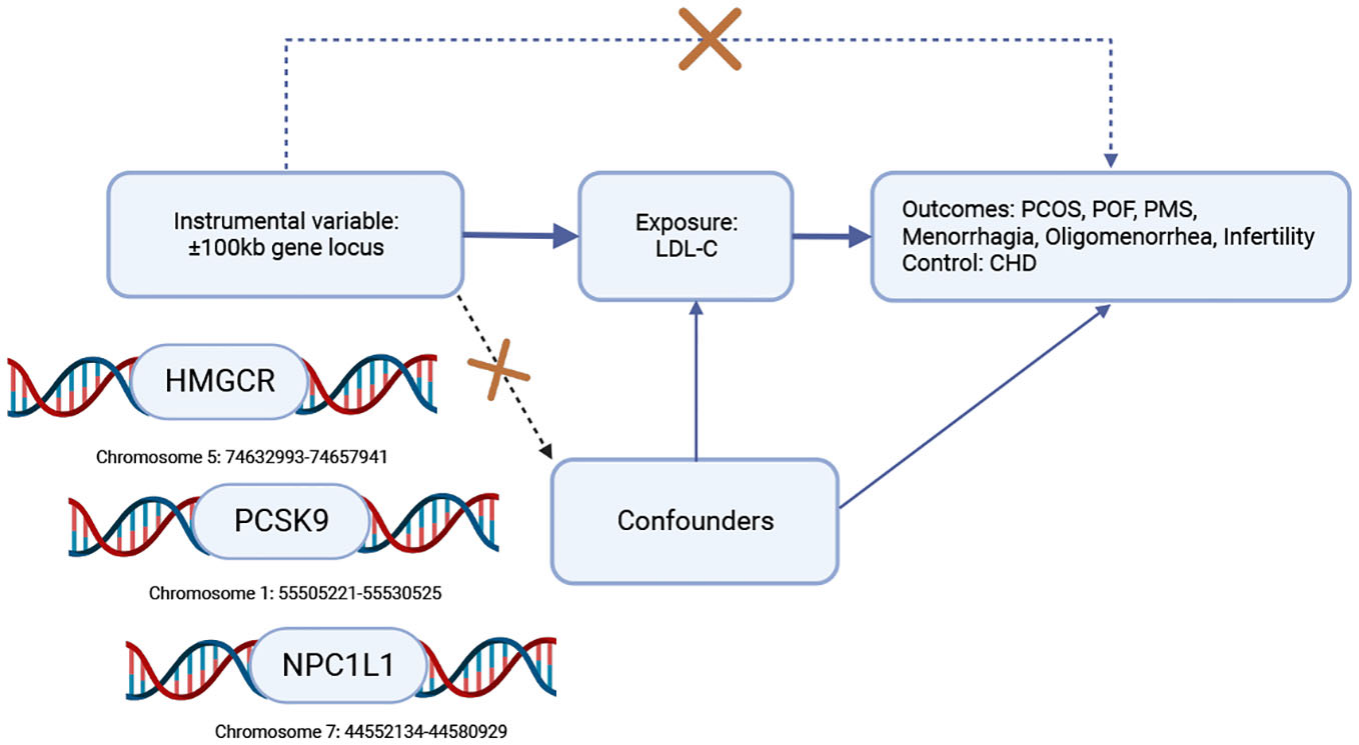
Workflow diagram for Mendelian randomization analysis of drug targets. To investigate the causal association, we selected coronary heart disease (CHD) as a positive control, given the widespread use of HMGCR, PCSK9, and NPC1L1 inhibitors to reduce the risk of CHD. To establish this association, the following conditions must be met: (1) the instrumental variable should not be associated with confounding factors (represented by dashed lines), (2) the instrumental variable should be strongly correlated with the exposure factor (represented by solid lines), and (3) the instrumental variable should not have a direct relationship with the outcome (represented by dotted lines). Key abbreviations used: LDL-C (low-density lipoprotein cholesterol), HMGCR (3-hydroxy-3-methylglutaryl-CoA reductase), PCSK9 (proprotein convertase subtilisin/kexin type 9), NPC1L1 (Niemann-Pick C1-Like 1), CHD (coronary heart disease), PCOS (polycystic ovary syndrome), POF (premature ovarian failure), PMS (premenstrual syndrome).

Exposure to lipid-lowering drugs was determined by selecting single nucleotide polymorphisms (SNPs) within a 100 kb window of each drug’s target gene that was correlated with LDL-C levels at a genome-wide significance level (MAF>1% (30) (**Supplementary File—Table 1**). The instrumental variables selected SNPs located within ±100kb of HMGCR, PCSK9, and NPC1L1 loci that were associated with LDL-C levels (**Figure 1** created with BioRender.com) while ensuring that the linkage disequilibrium was not too strong (r^2^ < 0.3). The Global Lipids Genetics Consortium (GLGC) data (ieu-a-300) retained 7 significant SNPs of HMGCR, 12 significant SNPs of PCSK9, and 3 SNPs of NPC1L1 (**Supplementary File — Table 2**), while the UK Biobank (UKB) data (ieu-b-110) retained 17 significant SNPs of HMGCR, 28 significant SNPs of PCSK9, and 6 SNPs of NPC1L1 (**Supplementary File—Table 3**).

### 2.2 Outcome data sources

We conducted an MR analysis of drug targets using data from 6 diseases, including PCOS, POF, PMS, menorrhagia, oligomenorrhea, and infertility. As a positive control dataset, we included CHD. The data sets for female reproductive endocrine diseases were obtained from the Finnish database R9 version (https://www.finngen.fi/en/access_results), with the majority of participants being of European descent. The CHD dataset was derived from GWAS summary statistics and comprised 60,801 cases and 123,504 controls (refer to **Supplementary File—Table 1**).

### 2.3 Data analysis

HMGCR, PCSK9, and NPC1L1 inhibitors have been extensively utilized in the treatment of CHD. To validate the instrumental variables, we employed the summary data from CHD GWAS as a positive control for our results. Initially, we aligned the drug-targeted instrumental variables with the outcome dataset and subsequently conducted analyses using various methods such as MR Egger, weighted median, inverse variance weighting (IVW), simple mode, weighted mode, and MR-PRESSO. The weighted mode clusters SNPs into groups based on the similarity of individual ratio estimates. This is done by calculating the cubic variance weight of the SNPs in each cluster and generating causal estimates based on the cluster with the highest SNP weight (31, 32). MR-Egger regression is a suitable method for evaluating data with pleiotropic effects (33), while weighted median analysis is significant for datasets with less than 50% of effective instrumental variables (34). The IVW method considered the most important method for MR analysis, further evaluates the impact of exposure factors on the results. It does this by calculating the Wald ratio of each effective SNP and then combining the Wald ratios using inverse variance as the weight (35, 36). Among these methods, IVW is the most commonly used approach (37), especially for the number of SNPs ≤3. In our study, the IVW method was deemed most suitable for analyzing our data, and therefore, we adopted the IVW analysis results. Heterogeneity testing was carried out using the MR Egger and IVW methods, where Cochrane’s Q value was utilized to assess the heterogeneity of genetic tools. A p-value > 0.05 indicated no significant heterogeneity. The MR Egger regression equation was employed to evaluate the horizontal pleiotropy of genetic tools, with a p-value > 0.05 indicating no horizontal pleiotropy (38). SNPs directly associated with the outcome were eliminated. Subsequently, we performed sensitivity analysis after removing outliers through the MR-PRESSO test. To ensure that our results were not significantly influenced by a specific SNP, we utilized the leave-one-out method to sequentially remove each SNP and compared the results using the IVW method. Data analysis was conducted using R version 4.2.2 with the MRPRESSO and TwoSampleMR software packages (38, 39). Finally, we merged the odds ratio (OR) values of the two datasets using the meta package.

## 3 Results

### 3.1 Positive control analysis

**Figure 2:**
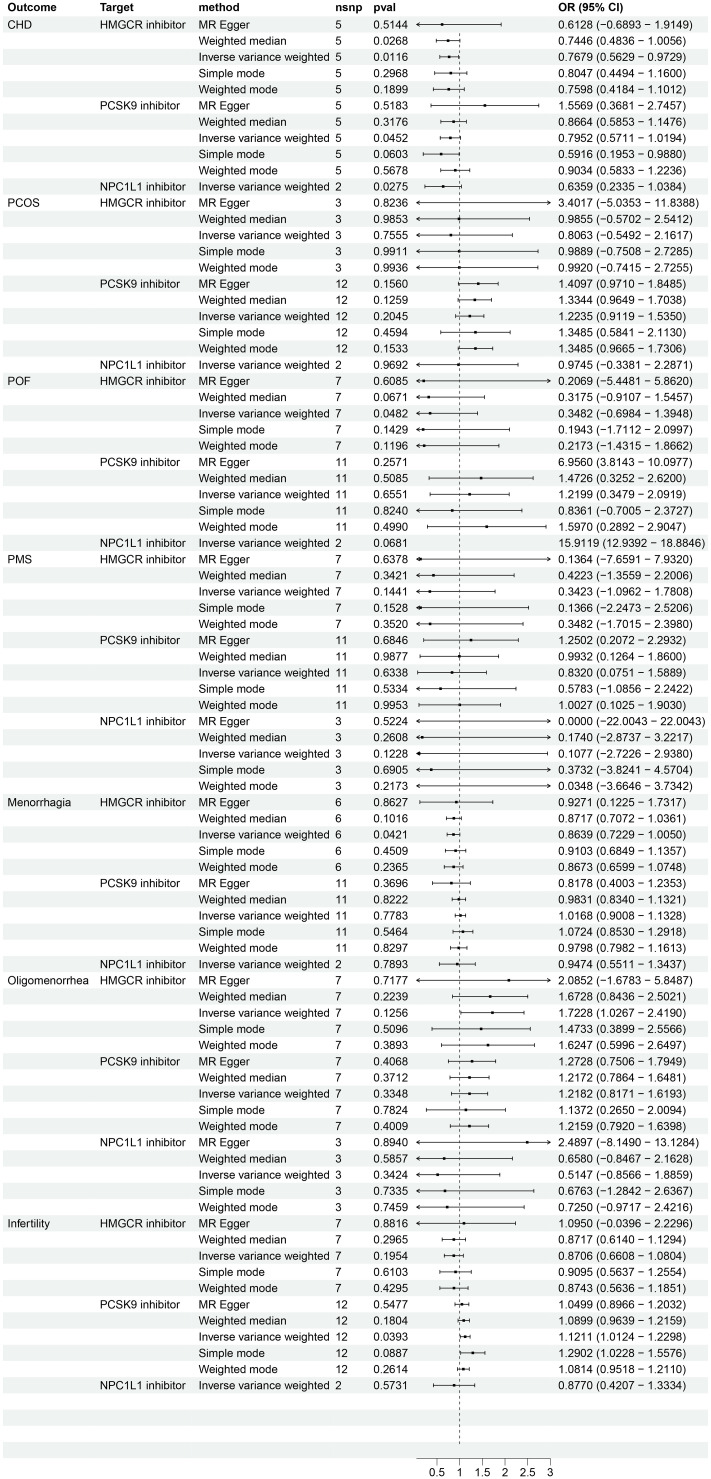
The effects of HMGCR, PCSK9, and NPC1L1 inhibitors on coronary heart disease and female reproductive endocrine diseases based on the exposure data derived from the GLGC (The Global Lipids Genetics Consortium) and identified as ieu-a-300 in the IEU OpenGWAS project. The abbreviations used are as follows: nsnp (number of single nucleotide polymorphisms), OR (odds ratio), CI (confidence interval), CHD (coronary heart disease), PCOS (polycystic ovary syndrome), POF (premature ovarian failure), and PMS (premenstrual syndrome). HMGCR refers to 3-hydroxy-3-Methylglutaryl-CoA reductase, PCSK9 stands for proprotein convertase subtilisin/kexin 9, and NPC1L1 represents Niemann-Pick C1-Like 1.

**Figure 3:**
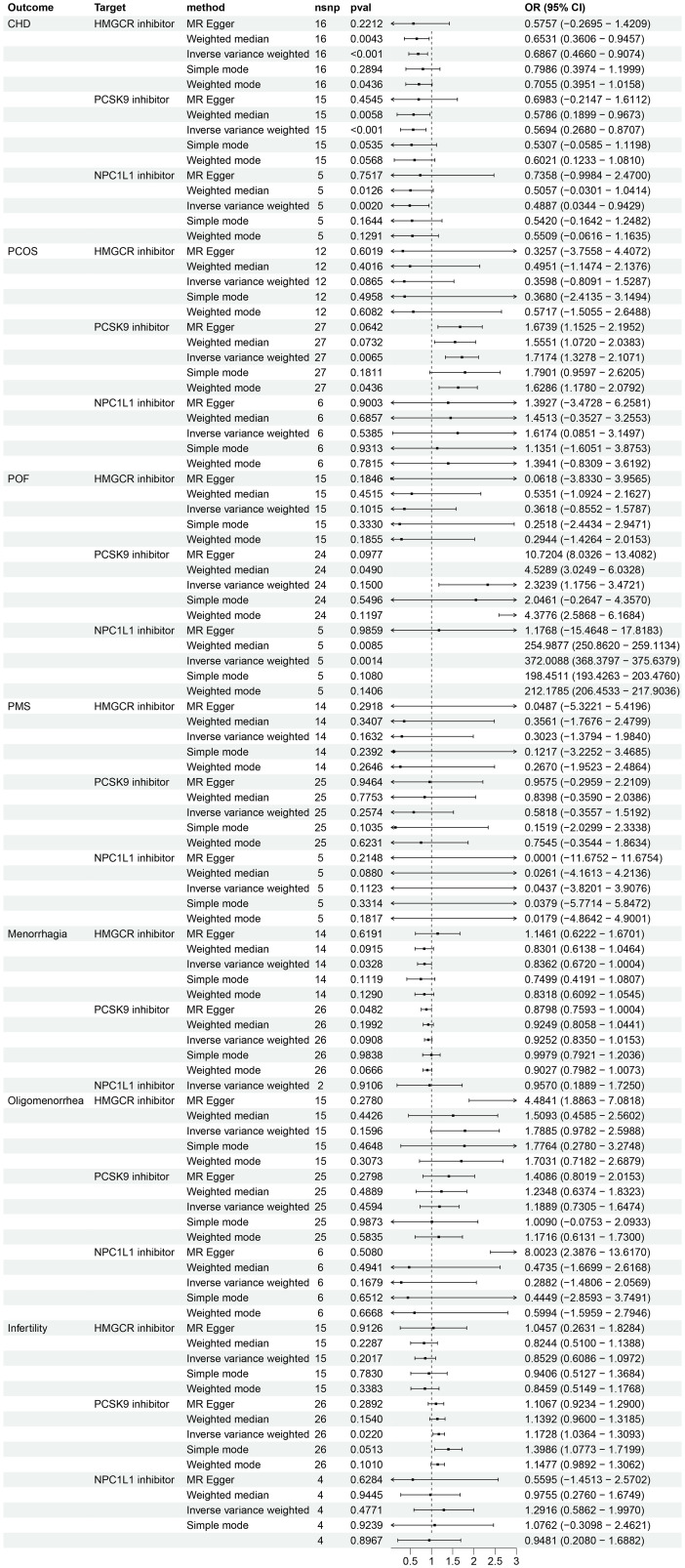
The effects of HMGCR, PCSK9, and NPC1L1 inhibitors on coronary heart disease and female reproductive endocrine diseases based on the exposure data derived from the UKB (The UK Biobank) and identified as ieu-b-110 in the IEU OpenGWAS project. The abbreviations used are as follows: nsnp (number of single nucleotide polymorphisms), OR (odds ratio), CI (confidence interval), CHD (coronary heart disease), PCOS (polycystic ovary syndrome), POF (premature ovarian failure), and PMS (premenstrual syndrome). HMGCR refers to 3-hydroxy-3-Methylglutaryl-CoA reductase, PCSK9 stands for proprotein convertase subtilisin/kexin 9, and NPC1L1 represents Niemann-Pick C1-Like 1.

The IVW method was used to analyze GLGC data, which indicated that HMGCR inhibitor (HMGCRi), PCSK9 inhibitor (PCSK9i), and NPC1L1 inhibitor (NPC1L1i) have a significant potential to reduce the risk of CHD (OR [95%] = 0.7679 [0.5629, 0.9729], p = 1.16 × 10^−2^; OR [95%] = 0.7952 [0.5711, 1.0194], p = 4.52 × 10^−2^; OR [95%] = 0.6359[0.2335, 1.0384], p = 2.75 × 10^−2^) (as shown in **Figure 2**). Results from MR Egger, simple mode, weighted mode, and MR-PRESSO are also presented. Another GWAS data set from UKB replicated the analysis and obtained similar results (HMGCRi: OR [95%] = 0.6867 [0.4660, 0.9074], p = 8.46 × 10^−4^; PCSK9i: OR [95%] = 0.5694 [0.2680, 0.8707], p = 2.49 × 10^−4^; NPC1L1i: OR [95%] = 0.4887 [0.0343, 0.9429], p = 2.00 × 10^−3^) (**Figure 3**).

### 3.2 The causal relationship between HMGCR, PCSK9 and NPC1L1 gene mimic inhibitors and female reproductive endocrine diseases

Analysis using exposure data from GLGC found that HMGCR inhibitor was associated with a protective effect for POF (IVW: OR [95%CI] = 0.3482 [-0.6984, 1.3948], p = 4.82 × 10^−2^) (**Figure 2**). However, the analysis results of exposure data from the UKB did not show a statistically significant difference (**Figure 3**).

An analysis using exposure data from UKB revealed that PCSK9 inhibitor has the potential to significantly increase the risk of patients with PCOS (IVW: OR [95%CI] = 1.7174 [1.3278, 2.1071], p=0.65× 10^−2^; Weighted model: OR [95%CI] = 1.6286 [1.1780, 2.0792], p=4.36× 10^−2^). The weighted median analysis method indicated that PCSK9 inhibitor could be a risk factor for patients with POF (OR [95%CI] = 4.5289 [3.0249, 6.0328], p=4.90× 10^−2^). The results of the IVW analysis and weighted median analysis suggest that NPC1L1 inhibitor may also increase the risk of POF (IVW: OR [95%CI] = 372.0088 [368.3797, 375.6379], p=0.14× 10^−2^; Weighted median: OR [95%CI] = 254.9877 [250.8620, 259.1134], p=0.85× 10^−2^). The MR Egger method demonstrated that PCSK9 inhibitor has the potential to decrease the risk in patients with menorrhagia (OR [95%CI] = 0.8798 [0.7593, 1.0004], p=4.82× 10^−2^) (refer to **Figure 3**). However, the exposure data from GLGC did not show any significant difference in the aforementioned outcomes (refer to **Figure 2**).

**Figure 4:**
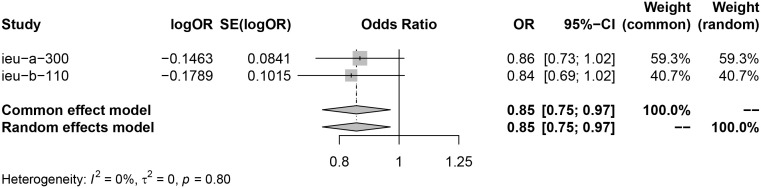
HMGCR inhibitors may reduce risk of menorrhagia by merging the OR value of two sets of data (exposure data from the GLGC and the UKB respectively). Key abbreviations used: ieu-a-300 (the exposure data from the GLGC), ieu-b-110(the exposure data from the UKB), OR (odds ratio), and CI (confidence interval). A P value of less than 0.05 indicates significant heterogeneity in the data.

**Figure 5:**
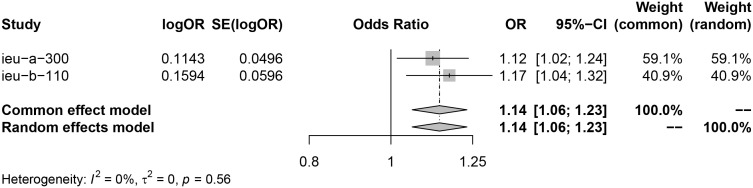
PCSK9 inhibitors may increase risk of infertility by merging the OR value of two sets of data (exposure data from the GLGC and the UKB respectively). Key abbreviations used: ieu-a-300(the exposure data from the GLGC), ieu-b-110 (the exposure data from the UKB), OR (odds ratio), and CI (confidence interval). A P value of less than 0.05 indicates significant heterogeneity in the data.

Analysis using exposure data from GLGC found that PCSK9 inhibitor was associated with a higher risk of infertility in patients (IVW: OR [95%CI] = 1.1211 [1.0124, 1.2298], p=3.93× 10^−2^). On the other hand, HMGCR inhibitor showed a significant reduction in the risk of menorrhagia in female patients (IVW: OR [95%CI] = 0.8639 [0.7229, 1.0050], p=4.21× 10^−2^) (**Figure 2**). These findings were further confirmed through analysis of exposure data from the UK Biobank, where the IVW analysis results were as follows: PCSK9 inhibitors - OR [95%CI] = 1.1728 [1.0364, 1.3093], p=2.20× 10^−2^; HMGCR inhibitors - OR [95%CI] = 0.8362 [0.6720, 1.0004], p=3.28× 10^−2^) (**Figure 3**). The meta package was utilized to combine the OR values, revealing that the HMGCR inhibitor was found to be a protective factor against menorrhagia in female patients (OR [95%CI] = 0.85 [0.75, 0.97]) (**Figure 4**). Conversely, PCSK9 inhibitors can increase the risk of infertility in patients (OR [95%CI] = 1.14 [1.06, 1.23]) (**Figure 5**).

### 3.3 Sensitivity analysis

To assess heterogeneity and horizontal pleiotropy, we utilized Cochrane’s Q and MR Egger regression equations (38) (**Additional File: Tables 4, 5**). After removing the SNPs that had a significant impact on the outcome in the leave-one-out analysis (**Supplementary Figure 1** and **Supplementary Figure 2**), the re-analysis results indicated no significant heterogeneity and horizontal pleiotropy. Therefore, we employed the fixed-effects IVW method for further analysis. However, it is important to note that the reliability of the GLGC data is compromised due to the limited number of SNPs identified in NPC1L1.

## 4 Discussion

Through a large-scale MR analysis of female reproductive endocrine diseases using data from a Finnish database, we investigated the effects of three common drug targets (HMGCR, PCSK9, and NPC1L1) on various conditions including PCOS, POF, PMS, menorrhagia, oligomenorrhea, and infertility. Our findings indicate that HMGCR inhibitors can reduce the risk of menorrhagia in women, but do not have a significant effect on patients with oligomenorrhea. On the other hand, PCSK9 inhibitors can significantly increase the risk of infertility in patients. It is worth noting that lipid-lowering drugs have not shown any effect on other female reproductive endocrine diseases, such as PCOS, POF, PMS and oligomenorrhea. These results suggest that statins (targeting HMGCR) may provide some protective effect against menorrhagia caused by uterine fibroids, adenomyosis, or functional abnormalities, while monoclonal antibodies like evolocumab (targeting PCSK9) may increase the risk of infertility.

According to the two-cell-two-gonadotropin theory (1), alterations in cholesterol levels can potentially impact the onset and progression of female reproductive diseases by influencing the actions of sex hormones. Studies have shown that statins are more effective at lowering LDL-C levels compared to hormone replacement therapy (40). Additionally, statins have similar effects on HDL cholesterol levels as hormone replacement therapy (40). While statins reduce triglyceride levels, hormone replacement therapy increases triglycerides (40). Apart from their cholesterol-lowering effects, statins have been found to exhibit anti-proliferative properties against certain tumors, including breast cancer (41), ovarian cancer (42), and colon cancer (43). A case-control study conducted within a cohort of female patients aged 18-65 years with hyperlipidemia revealed that the use of statins can reduce complications like menorrhagia and anemia caused by uterine fibroids (44). Our findings also suggest that HMGCR inhibitors, but not PCSK9 or NPC1L1 inhibitors, have a protective effect on patients with menorrhagia. This implies that the reduction in the occurrence of menorrhagia by HMGCR inhibitors is not only due to lowering LDL-C levels, but may also involve other protective mechanisms in patients with menorrhagia. This protective effect could be due to their anti-proliferative properties. However, since menorrhagia can have multiple causes, it cannot be ruled out that statins may also have an impact on menorrhagia caused by adenomyosis, functional uterine bleeding, and other factors. Further studies with more rigorous designs are needed to investigate this beyond uterine fibroids.

The interaction between statins and immunosuppressive drugs, especially calcineurin inhibitors, can limit the dosage or result in intolerance to this important class of drugs (45). Meanwhile, under-dosing or discontinuation of statin therapy can induce hyperlipidemia (46). A comprehensive meta-analysis of PCSK9 inhibitors has confirmed the drug’s safety profile, including its effects on neurocognitive adverse events, myalgia, new onset or worsening of pre-existing diabetes, elevated levels of creatine kinase, and the increase in alanine or aspartate aminotransferase having no significant impact (47). Apart from its impact on LDL-C levels, PCSK9 inhibitor also exhibits potential pleiotropic effects such as enhancing tumor response to immune checkpoint therapy, inhibiting platelet activation and thrombosis, and reducing apoptosis (18, 48, 49). In our study, it was observed that PCSK9i may increase the risk of infertility, while HMGCRi and NPC1L1i did not have similar effects. This suggests that PCSK9i may elevate the risk of infertility through pathways other than lipid lowering. Both alirocumab and evolocumab are monoclonal antibodies, which raises concerns about their potential antigenicity and adverse immune reactions. Our research findings indicate that PCSK9 inhibitors may pose a risk for infertility patients. Infertility could be attributed to adverse immune responses mediated by antibodies. However, further studies are needed to confirm this. Based on our study results, we recommend considering alternative lipid-lowering drugs instead of PCSK9 inhibitors for therapeutic purposes in female patients who require lipid-lowering treatment and have reproductive needs.

Cholesterol serves as the precursor for steroid hormones, and mevalonate plays a crucial role in the biosynthesis of cholesterol. The rate-limiting step in this process involves the conversion of 3-hydroxy-3-methylglutaryl coenzyme A (HMG-CoA) to mevalonate, which is facilitated by HMGCR. Research conducted on rodent ovarian theca-stromal cells has demonstrated that statins can effectively reduce the production of ovarian androgens (50). Additionally, studies have revealed that the addition of simvastatin to oral contraceptives can further decrease circulating testosterone (T) levels in women with PCOS (51, 52). Xu et al.’s study has also indicated that genetic variations in the HMGCR gene are associated with insulin resistance, sex hormone-binding globulin, and free testosterone levels in PCOS patients. The data on the effects of lipid-lowering drugs on PCOS and POF are not corroborated by exposure data from GLGC and UKB, and there is a possibility of errors in the experimental results. This difference could be attributed to the limitations of MR, which can only reflect the effects of lifetime exposure and cannot explore potential changes without continuous assessment. Therefore, while MR targeting drugs can provide insights into the direction of action, it may not directly predict the extent of a drug’s pharmacological effect on its target.

Research on blood lipids in reproductive endocrine diseases is often hindered by interference from hormone therapy and other conditions. However, Mendelian randomization studies offer clear advantages in studying the causal association and lifetime exposure risks between the two at the genetic level. Given the therapeutic value of lipid-lowering drugs in treating various diseases and the close connection between cholesterol and female hormones, it is important to investigate the long-term impact of these drugs on female reproductive endocrine health. Current clinical guidelines recommend the use of lipid-lowering drugs for women with female reproductive endocrine diseases accompanied by dyslipidemia. Nevertheless, it remains uncertain whether taking lipid-lowering drugs can affect the risk of reproductive endocrine diseases. Therefore, studying the influence of lipid-lowering drugs on lifelong exposure to reproductive endocrine diseases is of great significance. This research can help identify new treatment methods and strike a balance between the adverse effects of hormone therapy drugs on blood lipids during patient treatment. The findings of this study suggest that statins may be effective in reducing lipids in female patients with menorrhagia. However, caution should be exercised when considering the use of PCSK9 inhibitors to lower lipids in female patients who desire to conceive, as it may potentially increase the risk of infertility. It is important to acknowledge that our study has certain limitations. Firstly, it is important to note that MR analysis cannot replace clinical trials as it only provides a method for analyzing the causal relationship between exposure and outcome. Further research is required to confirm the association between HMGCR and PCSK9 inhibitors and their impact on menorrhagia and infertility. Secondly, our study only analyzed data from European populations due to limited GWAS data resources, which may limit the generalizability of our findings to other regions. Moreover, since it is GWAS summary data, stratified analysis cannot be conducted based on age or other factors. Additionally, the limited number of positive cases of gynecological endocrine diseases and the small dataset available may introduce potential data bias.

Following the drug-targeted MR analysis, our findings suggest that PCSK9 inhibitors are associated with a significant increase in the risk of infertility among patients. On the other hand, it appears that HMGCR inhibitors may have a protective effect against menorrhagia in women. However, no significant effects of lipid-lowering drugs have been observed on other reproductive endocrine disorders, such as PCOS.

## Data availability statement

The original contributions presented in the study are included in the article/**Supplementary Material**, further inquiries can be directed to the corresponding author/s.

## Author contributions

JZ: Investigation, Software, Writing – original draft, Writing – review & editing. RW: Investigation, Software, Writing – review & editing. LS: Writing – original draft. HH: Writing – original draft. PW: Writing – original draft. YaZ: Writing – original draft. YnZ: Writing – original draft. HZ: Writing – original draft.
