# Supplementary material for: Causal association between lipid-lowering drugs and female reproductive endocrine diseases: a drug-targeted Mendelian randomization study
Source: Front Endocrinol (Lausanne). 2023 Nov 10;14:1295412. doi: 10.3389/fendo.2023.1295412 (PMC10668027; doi:10.3389/fendo.2023.1295412)
Supplement: Supplementary file 2 [file DataSheet_1.pdf]

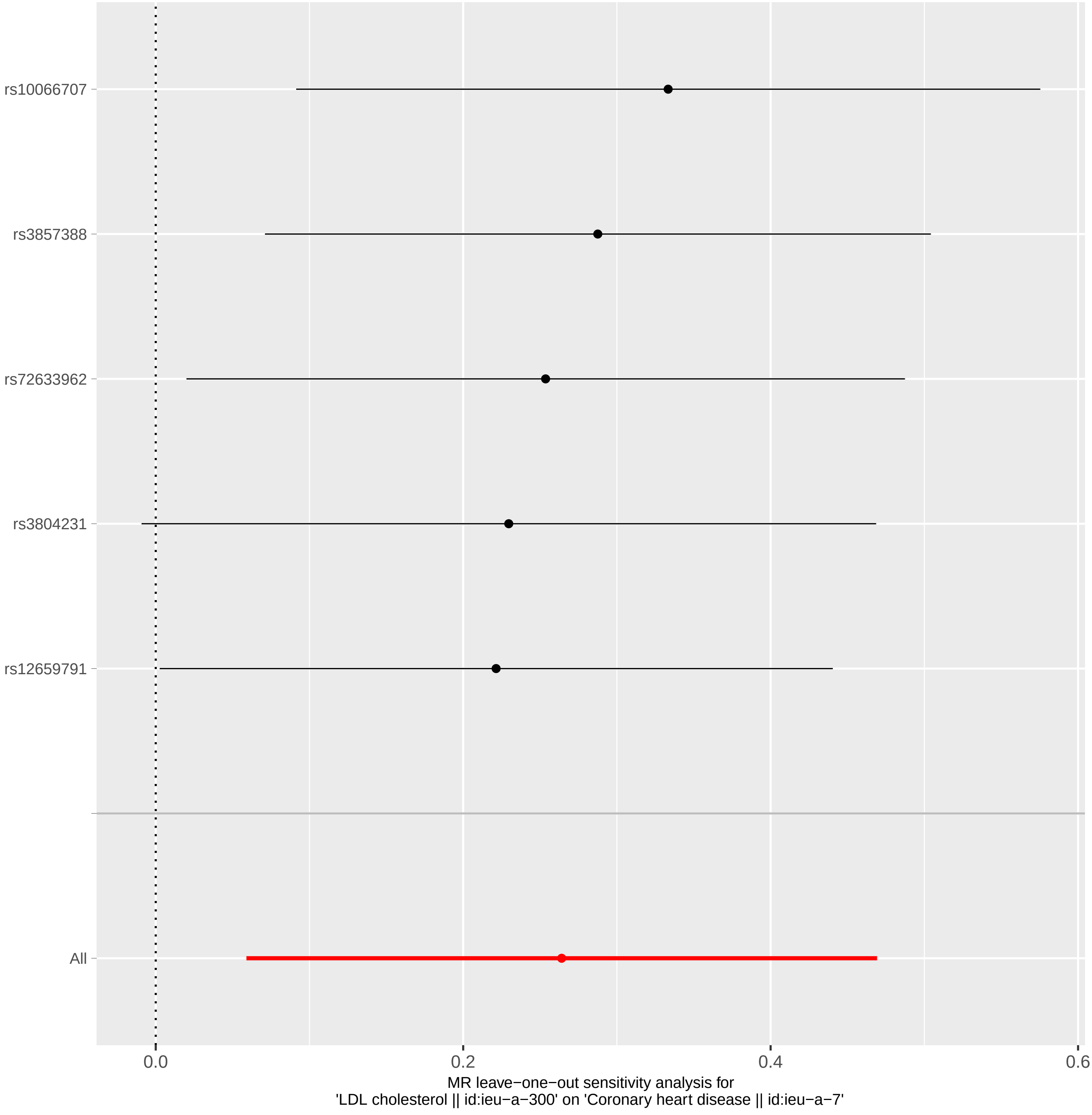
rs10066707
rs3857388
rs72633962
rs3804231
rs12659791
All
0.0
0.2
0.4
0.6
MR leave−one−out sensitivity analysis for
'LDL cholesterol || id:ieu−a−300' on 'Coronary heart disease || id:ieu−a−7'

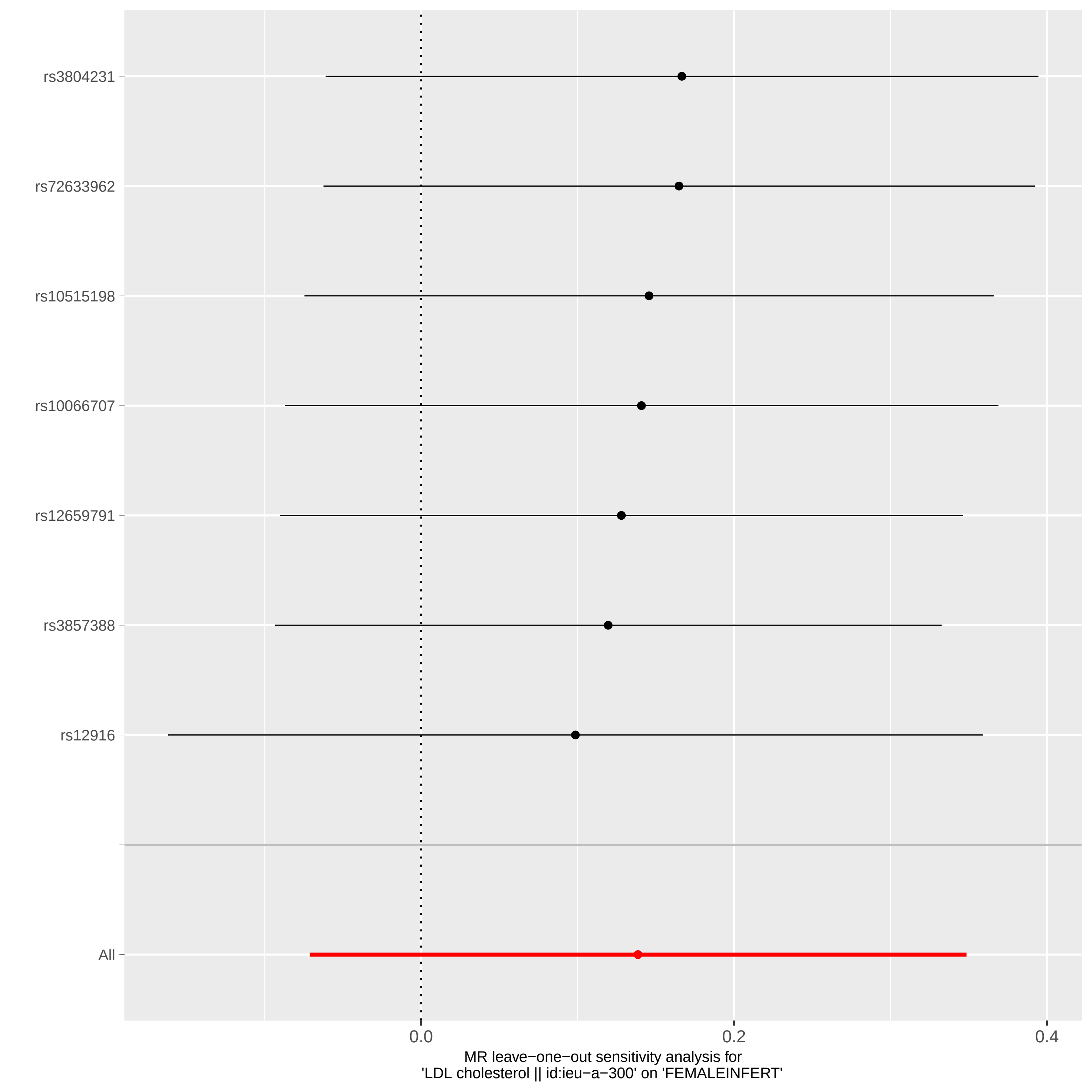
rs3804231
rs72633962
rs10515198
rs10066707
rs12659791
rs3857388
rs12916
All
0.0
0.2
0.4
MR leave−one−out sensitivity analysis for
'LDL cholesterol || id:ieu−a−300' on 'FEMALEINFERT'

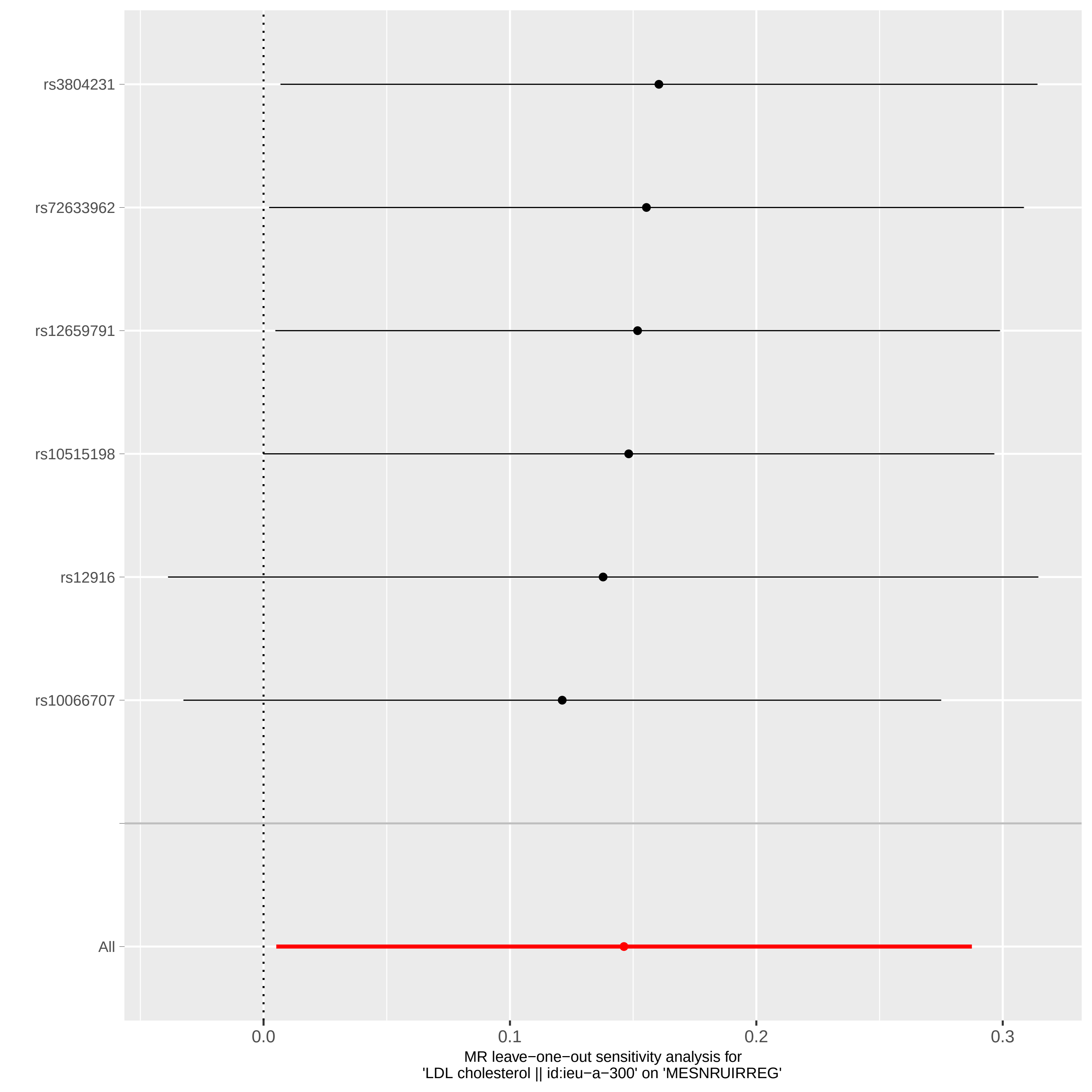

rs3804231
rs72633962
rs12659791
rs10515198
rs12916
rs10066707
All
0.0
0.1
0.2
0.3
MR leave−one−out sensitivity analysis for
'LDL cholesterol || id:ieu−a−300' on 'MESNRUIRREG'

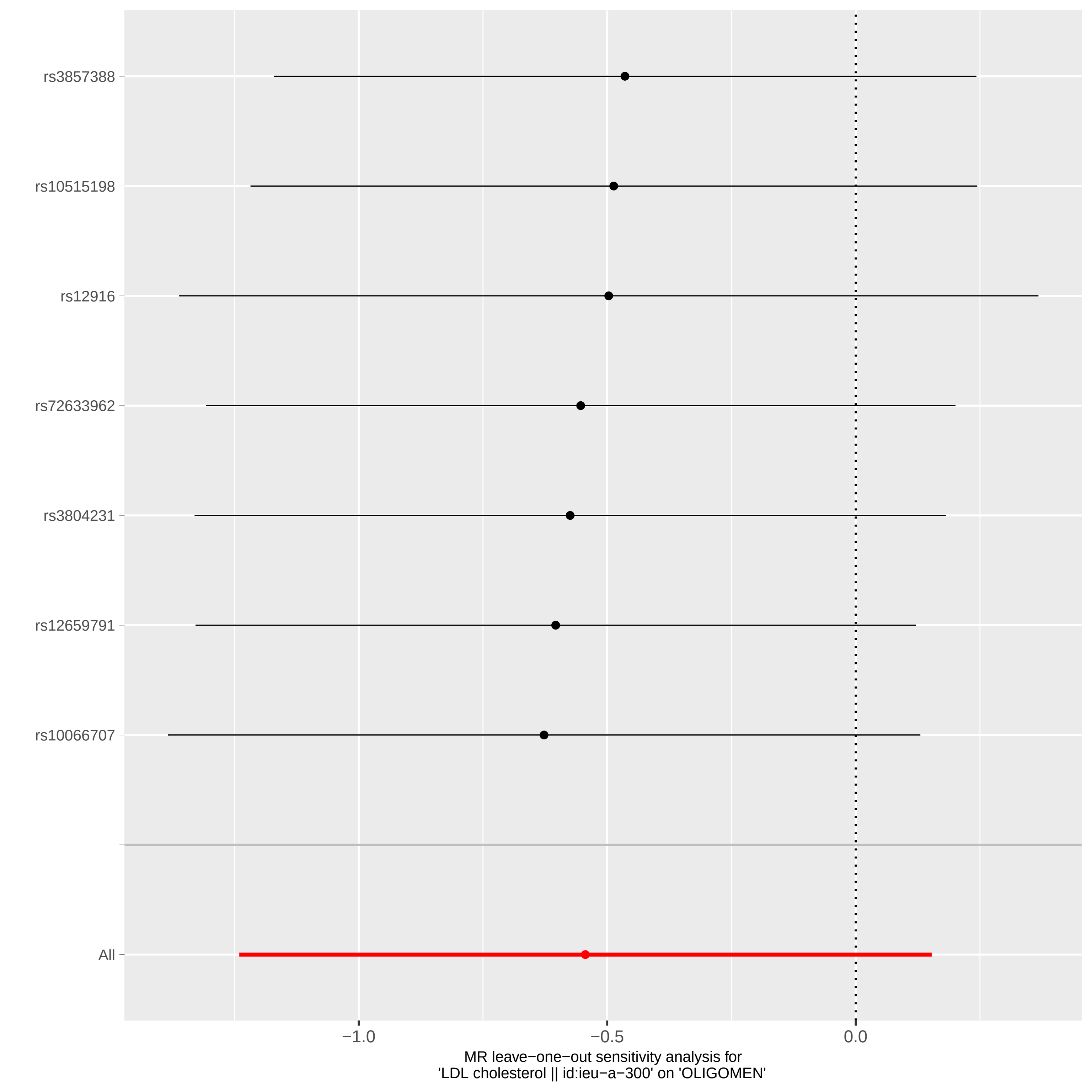
rs3857388
rs10515198
rs12916
rs72633962
rs3804231
rs12659791
rs10066707
All
−1.0
−0.5
0.0
MR leave−one−out sensitivity analysis for
'LDL cholesterol || id:ieu−a−300' on 'OLIGOMEN'

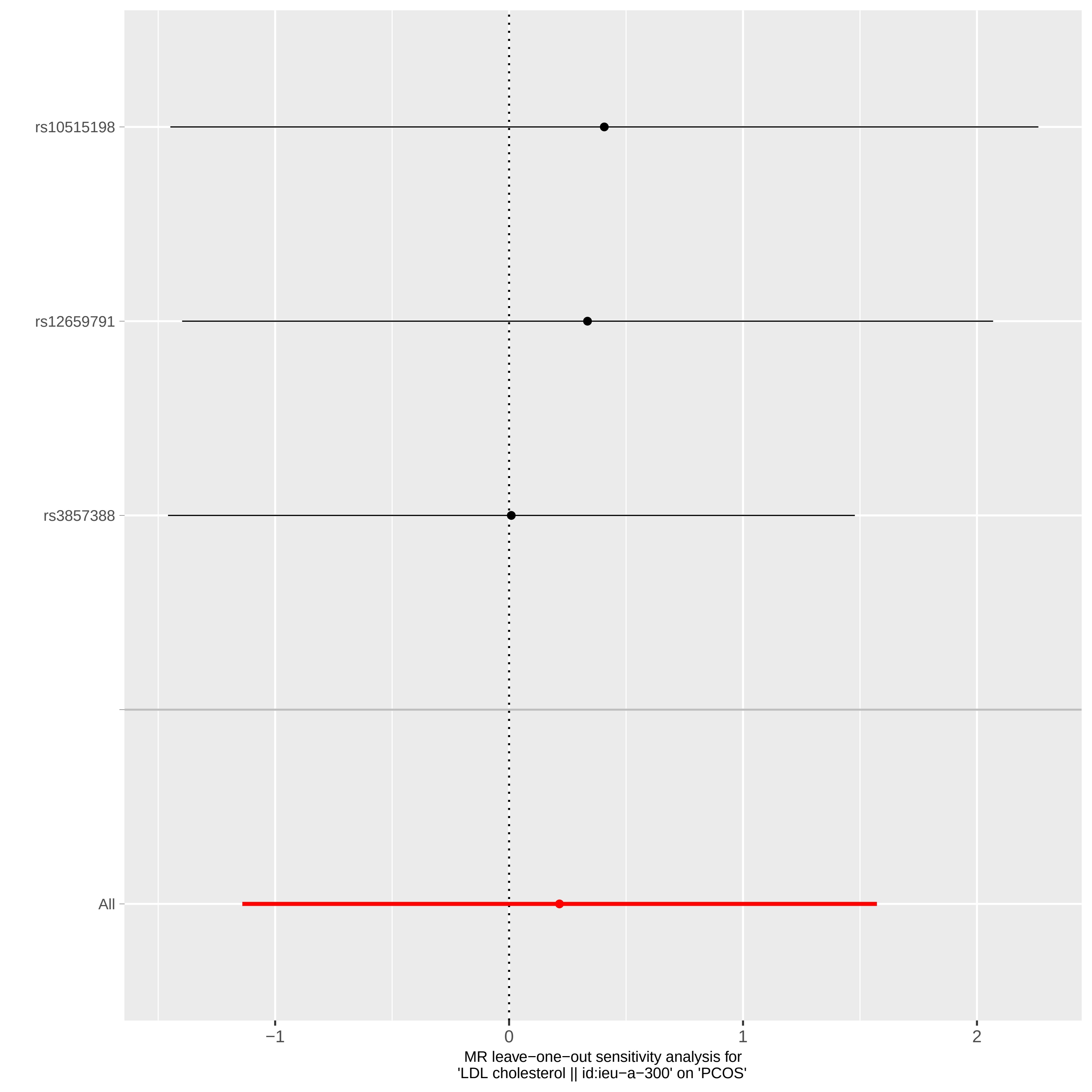
rs10515198
rs12659791
rs3857388
All
−1
0
1
2
MR leave−one−out sensitivity analysis for
'LDL cholesterol || id:ieu−a−300' on 'PCOS'

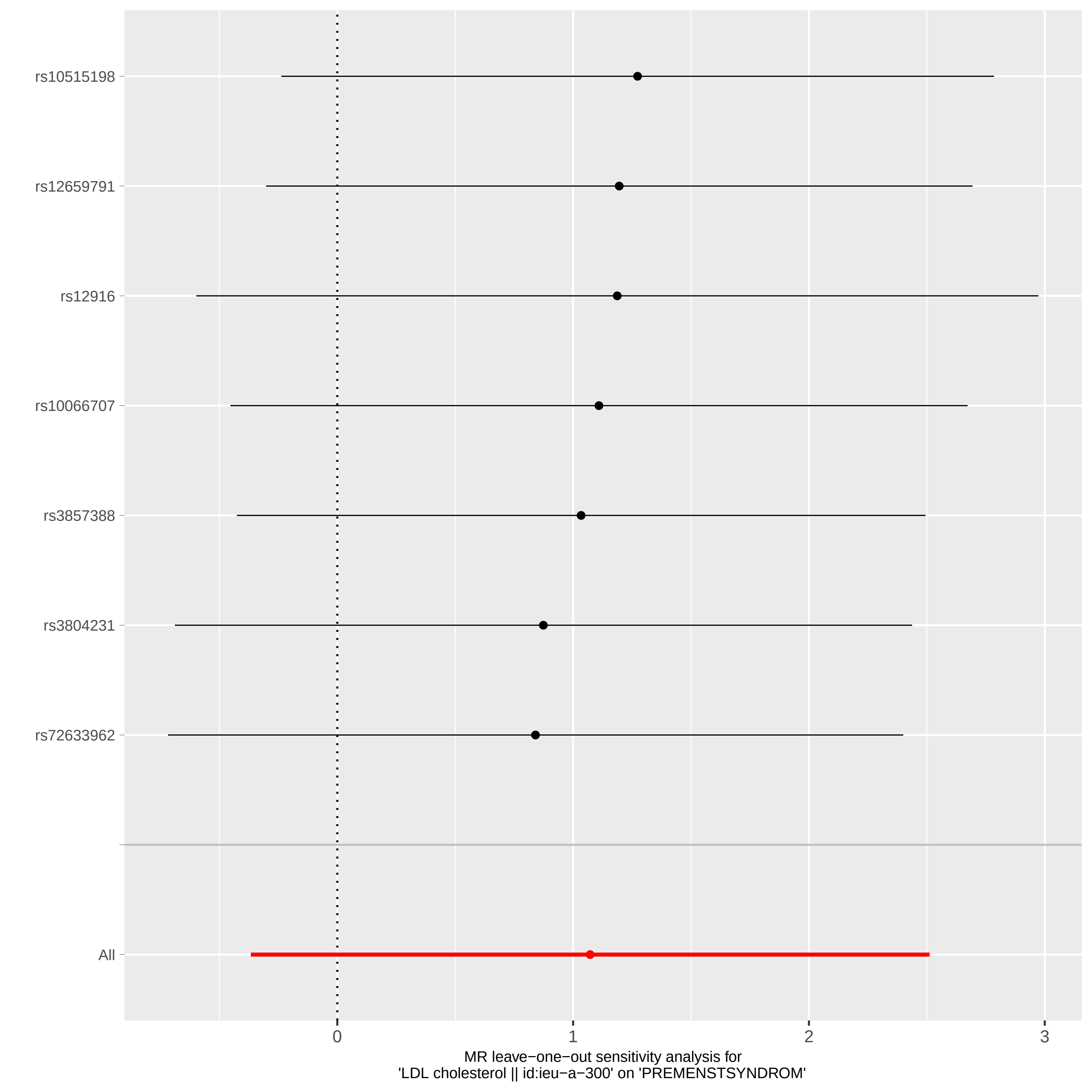

rs10515198
rs12659791
rs12916
rs10066707
rs3857388
rs3804231
rs72633962
All
0
1
2
3
MR leave−one−out sensitivity analysis for
'LDL cholesterol || id:ieu−a−300' on 'PREMENSTSYNDROM'

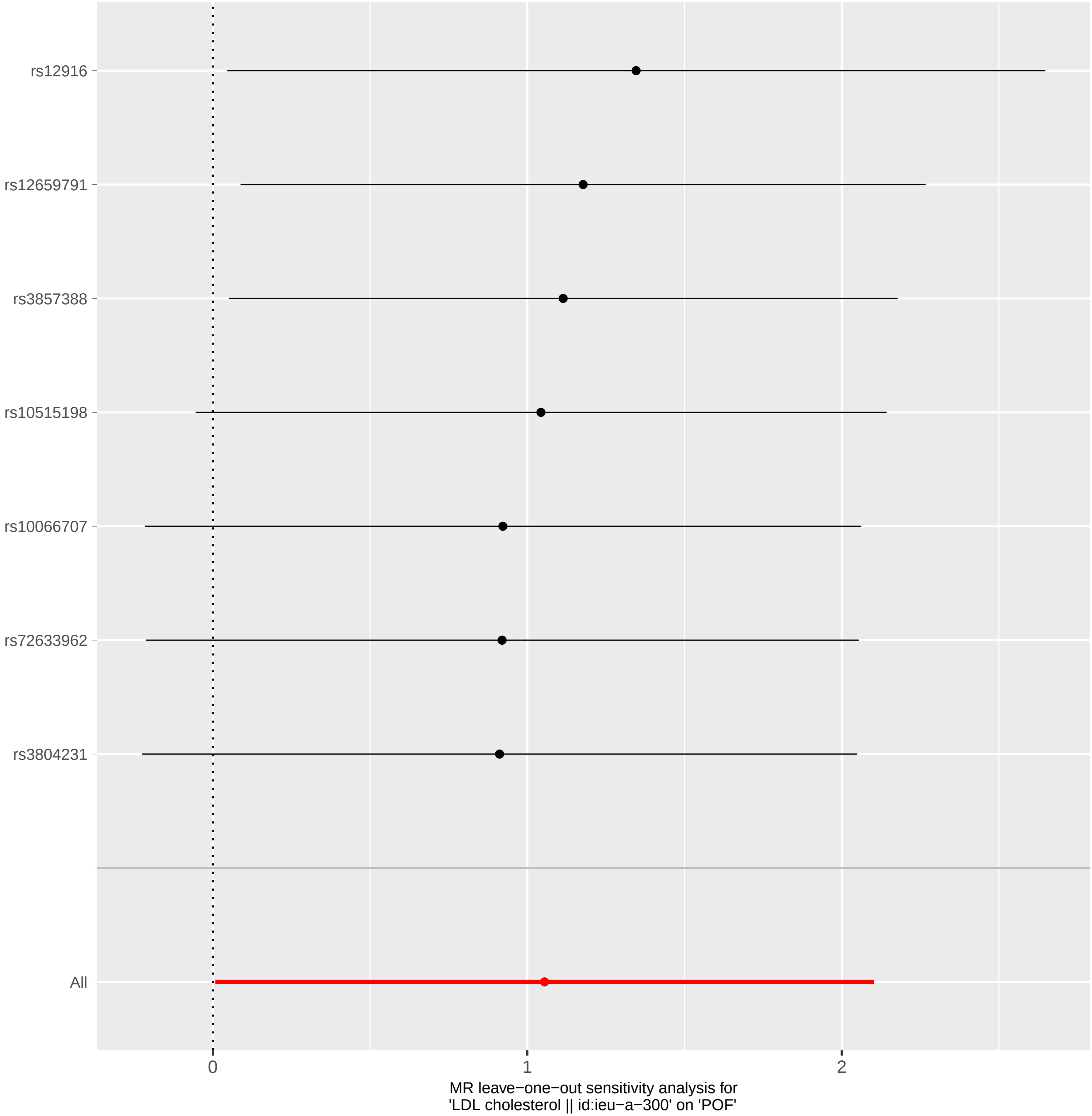

rs12916
rs12659791
rs3857388
rs10515198
rs10066707
rs72633962
rs3804231
All
0
1
2
MR leave−one−out sensitivity analysis for
'LDL cholesterol || id:ieu−a−300' on 'POF'

Insufficient number of SNPs

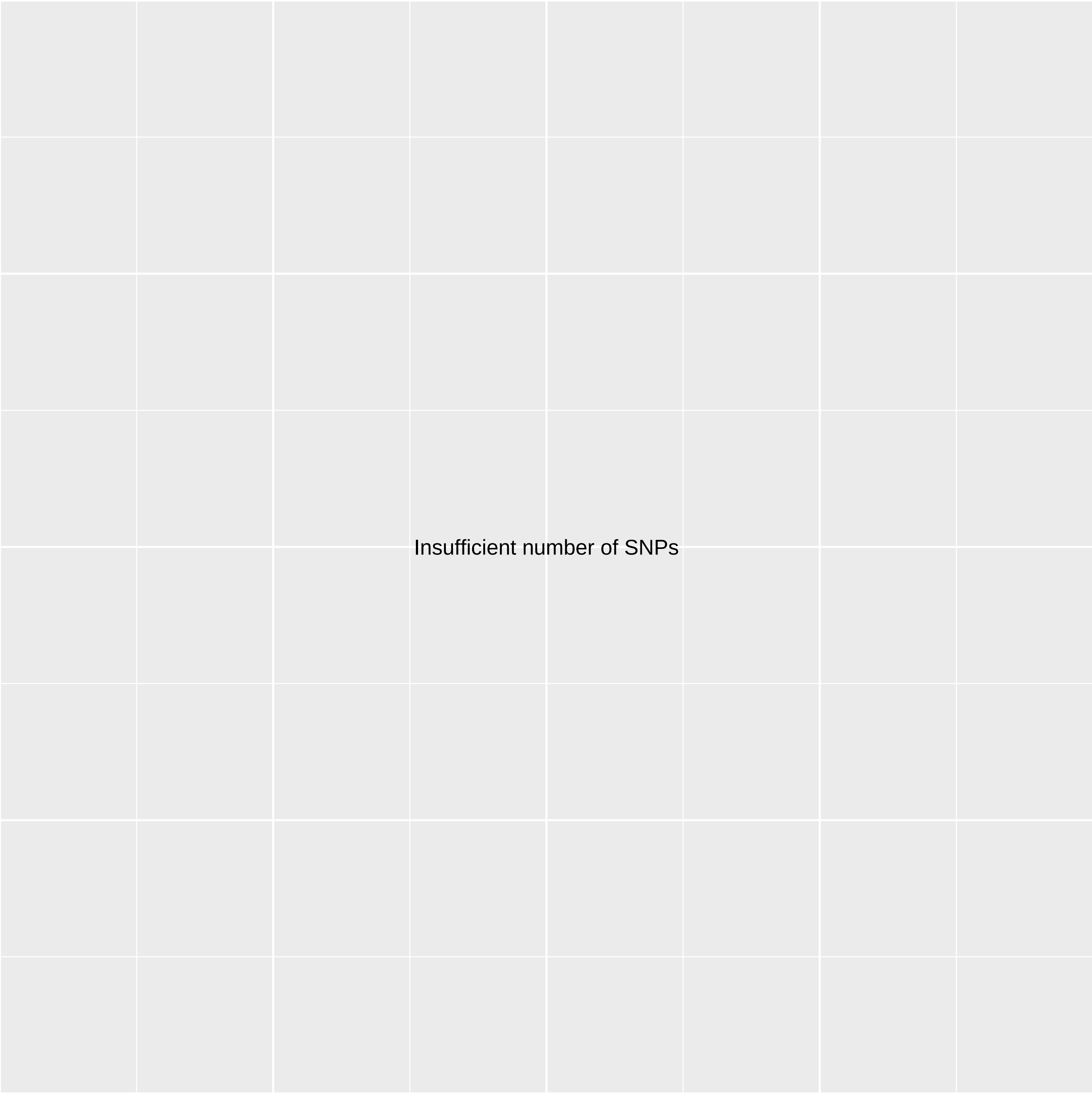
Insufficient number of SNPs

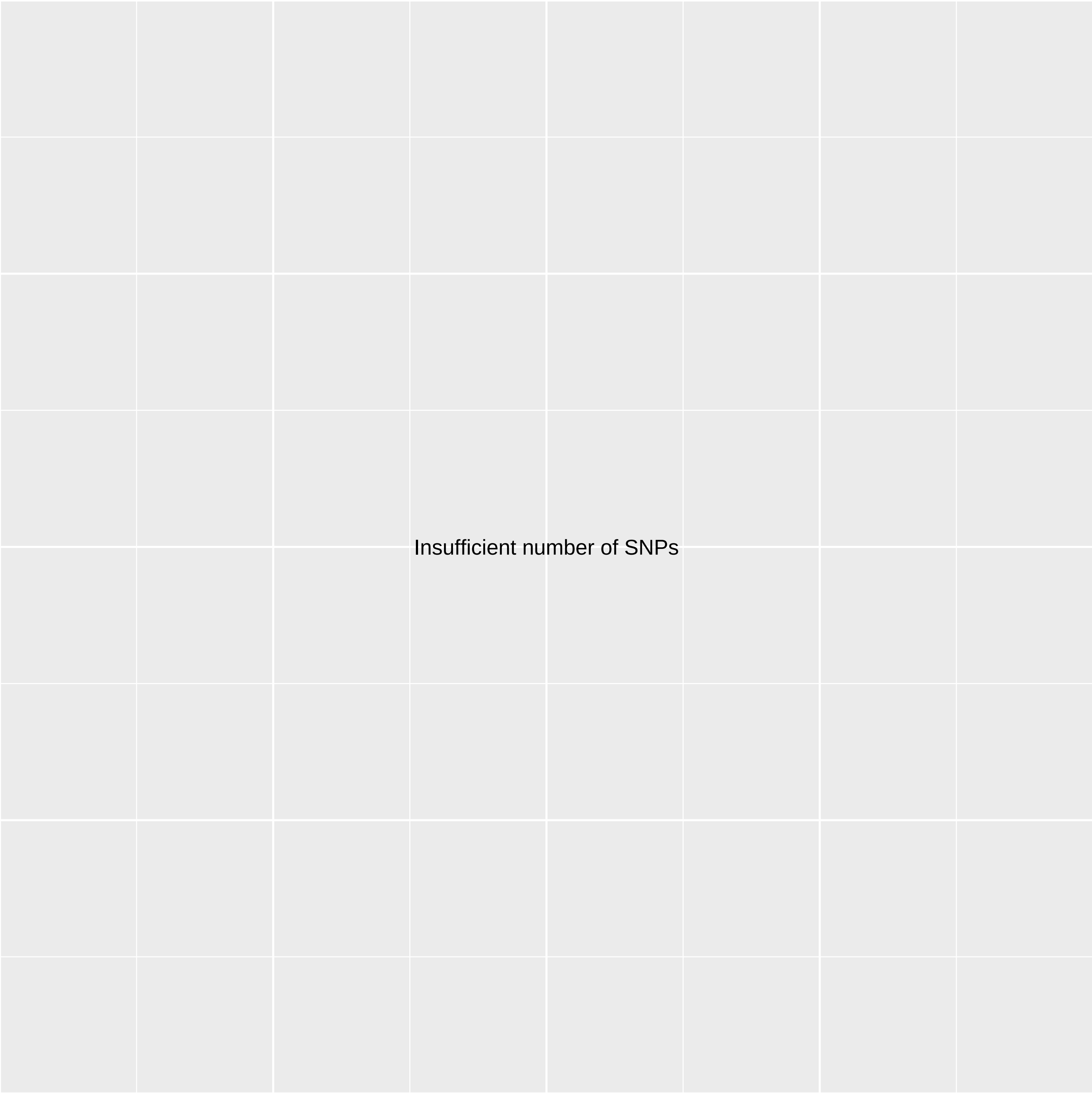
Insufficient number of SNPs

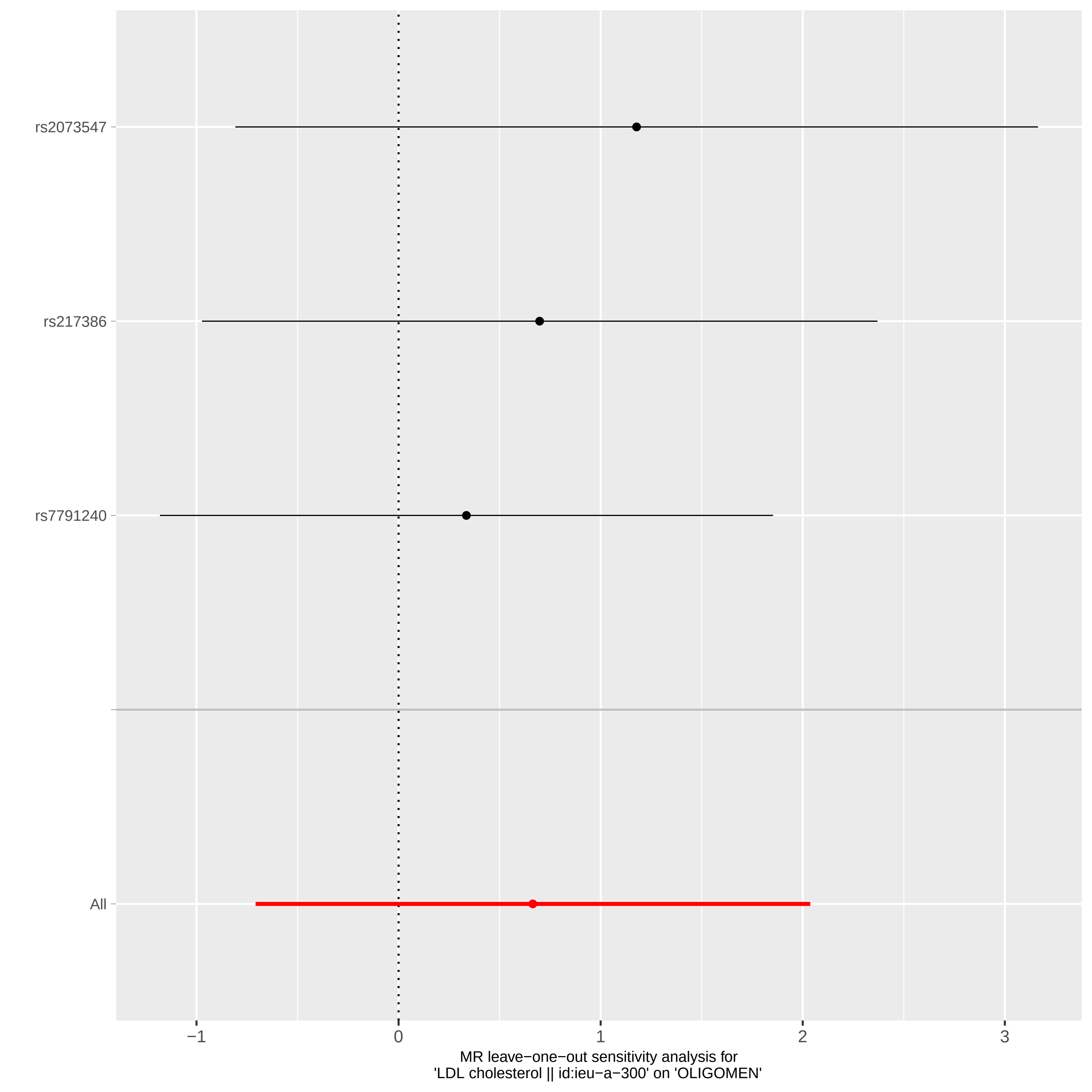
rs2073547
rs217386
rs7791240
All
−1
0
1
2
3
MR leave−one−out sensitivity analysis for
'LDL cholesterol || id:ieu−a−300' on 'OLIGOMEN'

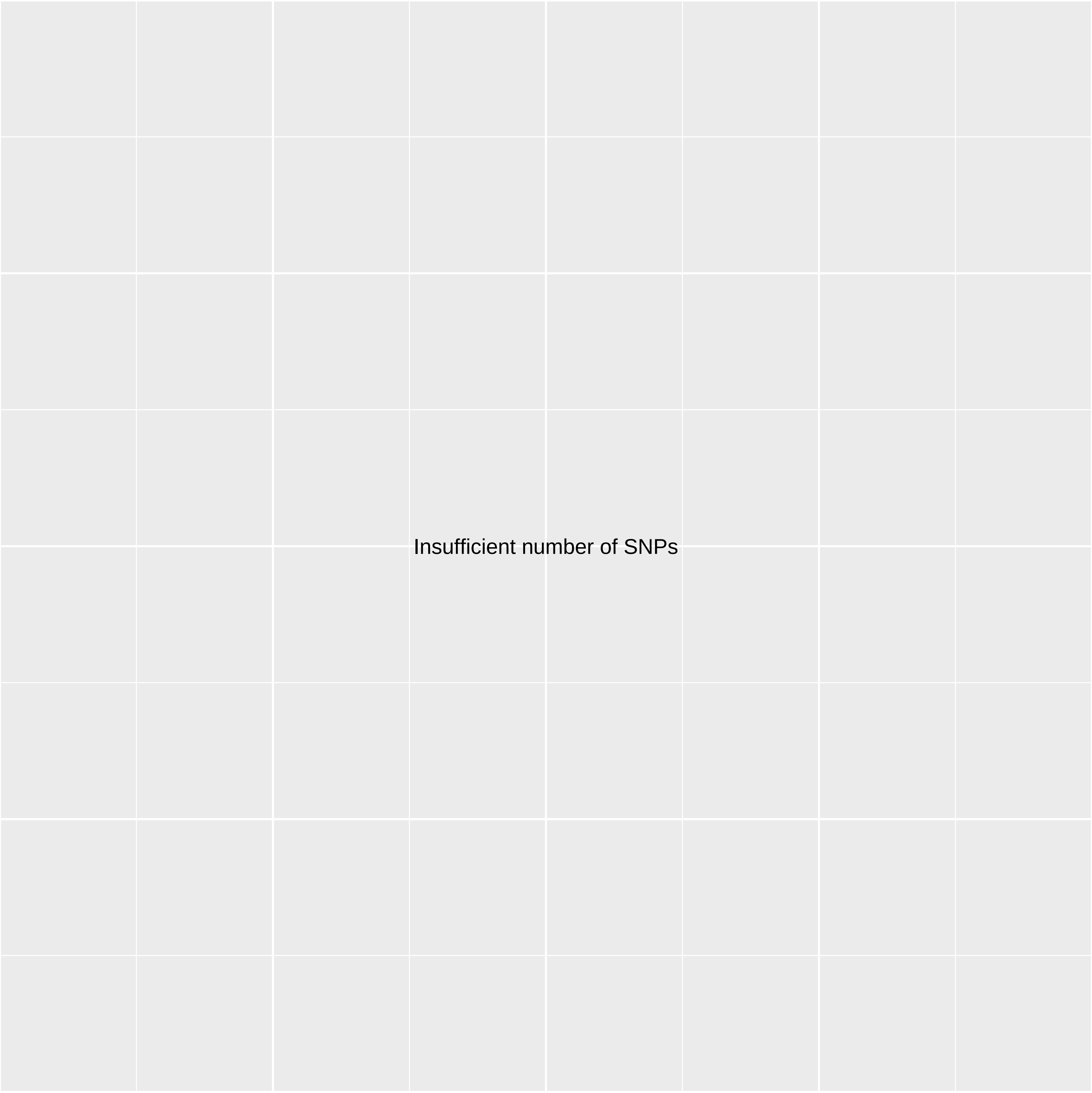
Insufficient number of SNPs

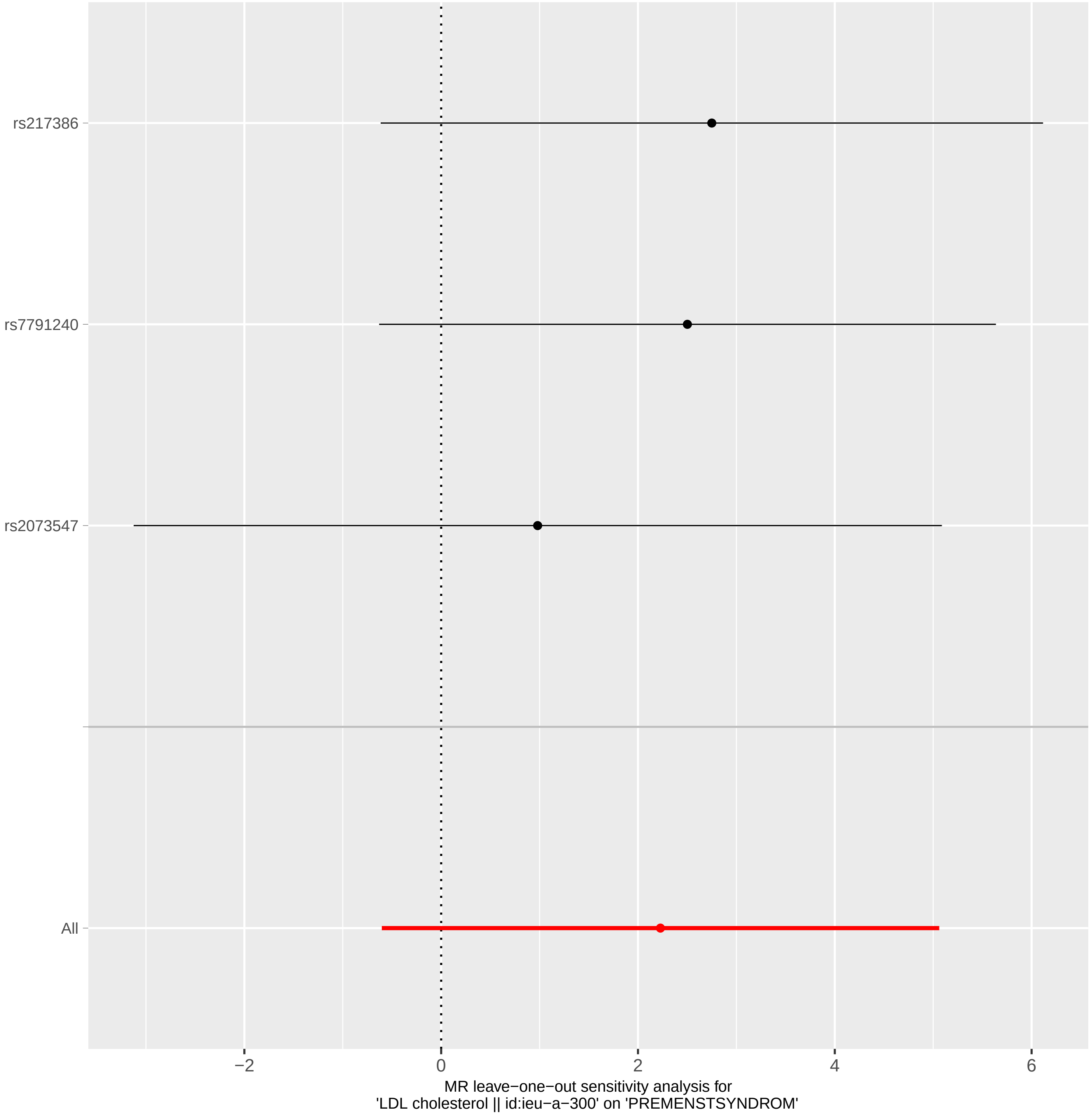
rs217386
rs7791240
rs2073547
All
−2
0
2
4
6
MR leave−one−out sensitivity analysis for
'LDL cholesterol || id:ieu−a−300' on 'PREMENSTSYNDROM'

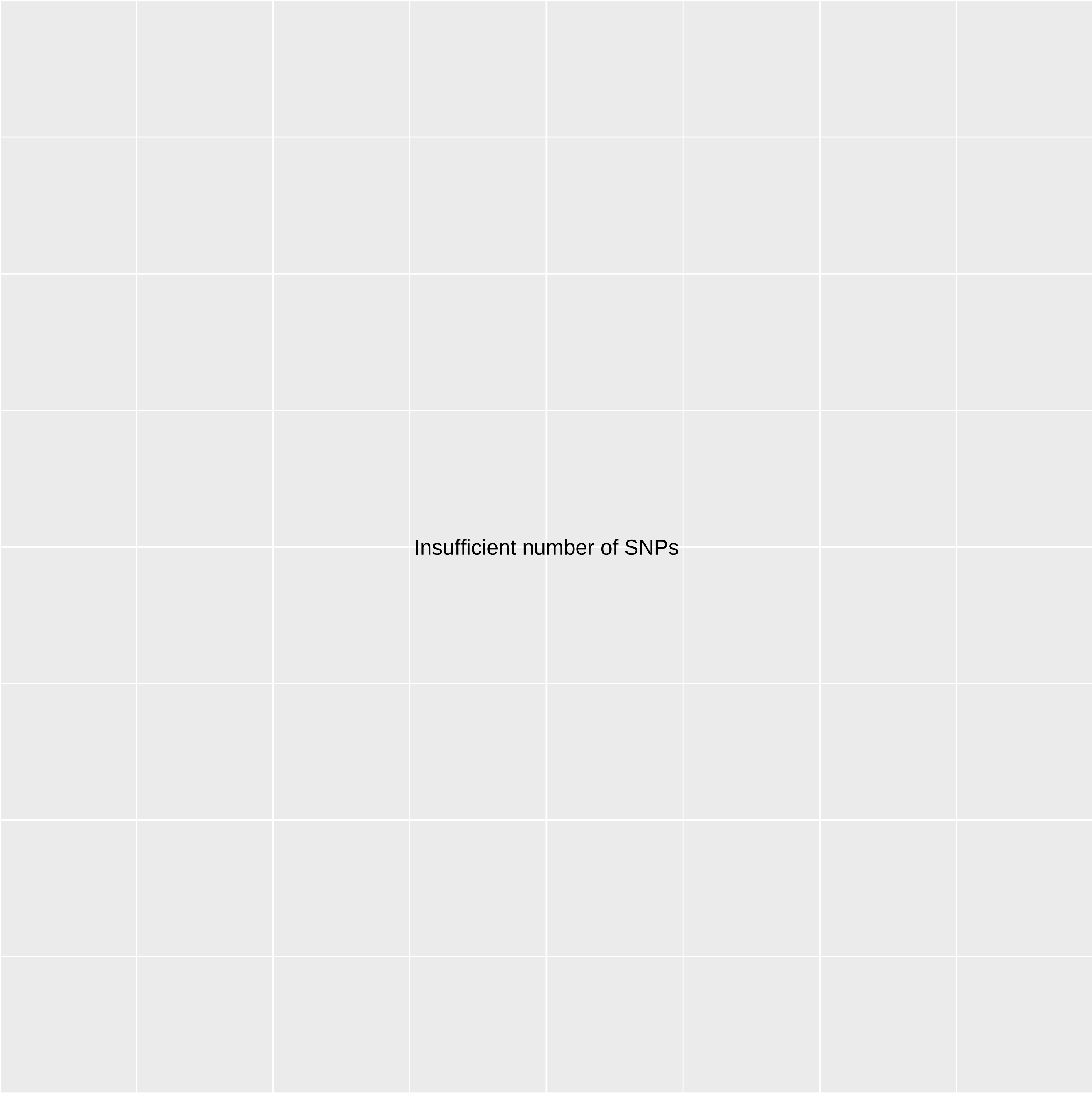
Insufficient number of SNPs

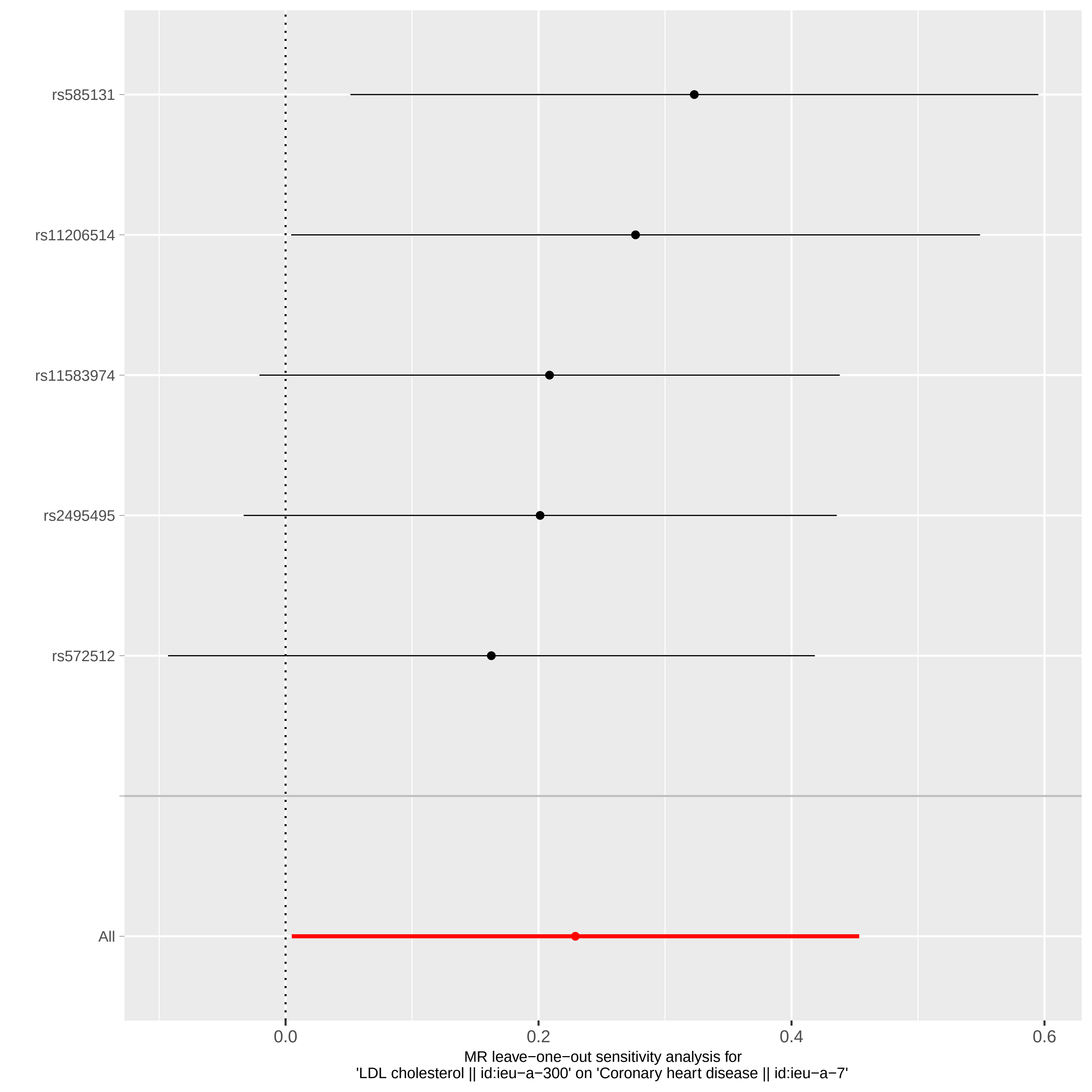
rs585131
rs11206514
rs11583974
rs2495495
rs572512
All
0.0
0.2
0.4
0.6
MR leave−one−out sensitivity analysis for
'LDL cholesterol || id:ieu−a−300' on 'Coronary heart disease || id:ieu−a−7'

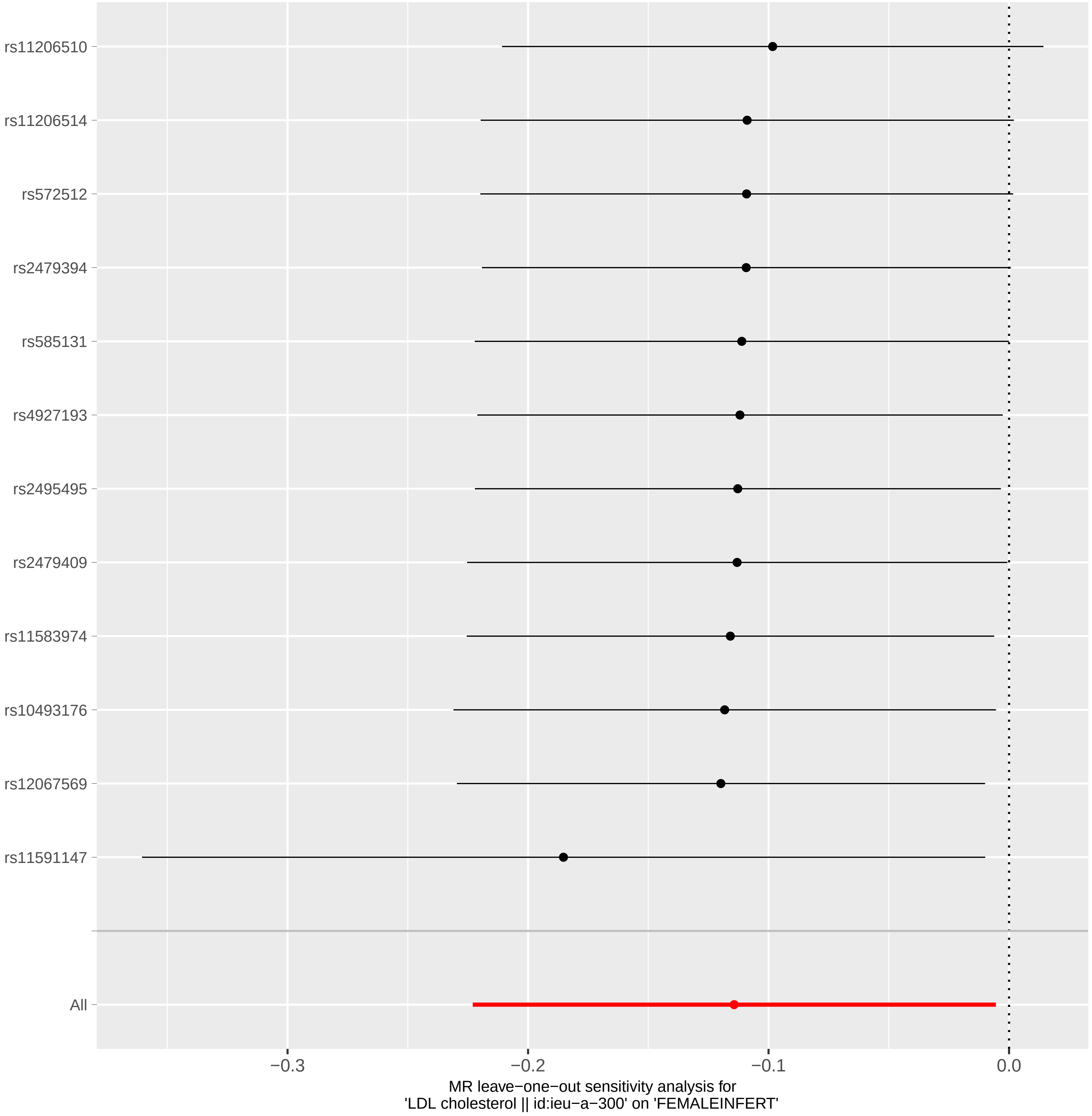
rs11206510
rs11206514
rs572512
rs2479394
rs585131
rs4927193
rs2495495
rs2479409
rs11583974
rs10493176
rs12067569
rs11591147
All
−0.3
−0.2
−0.1
0.0
MR leave−one−out sensitivity analysis for
'LDL cholesterol || id:ieu−a−300' on 'FEMALEINFERT'

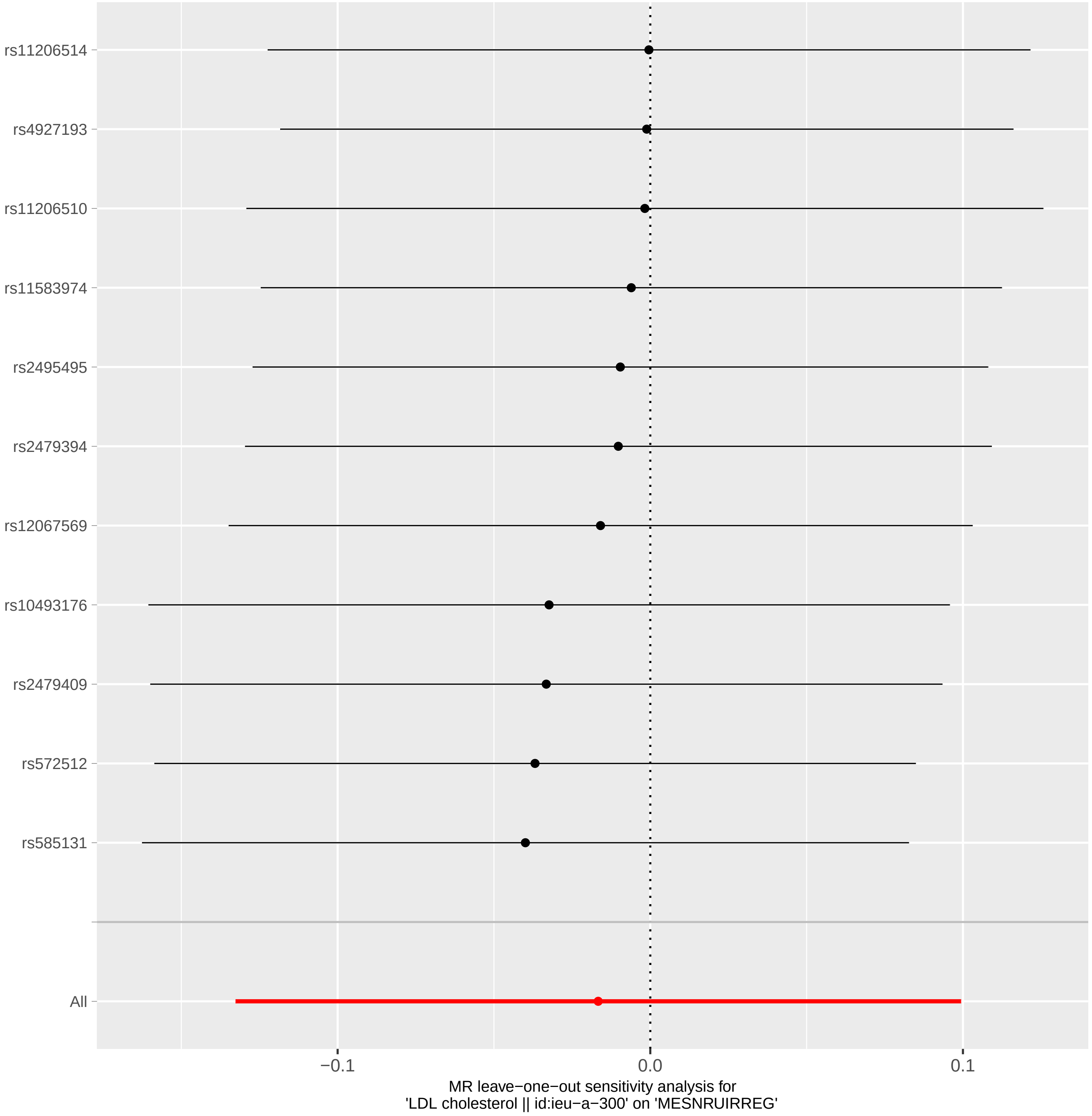
rs11206514
rs4927193
rs11206510
rs11583974
rs2495495
rs2479394
rs12067569
rs10493176
rs2479409
rs572512
rs585131
All
−0.1
0.0
0.1
MR leave−one−out sensitivity analysis for
'LDL cholesterol || id:ieu−a−300' on 'MESNRUIRREG'

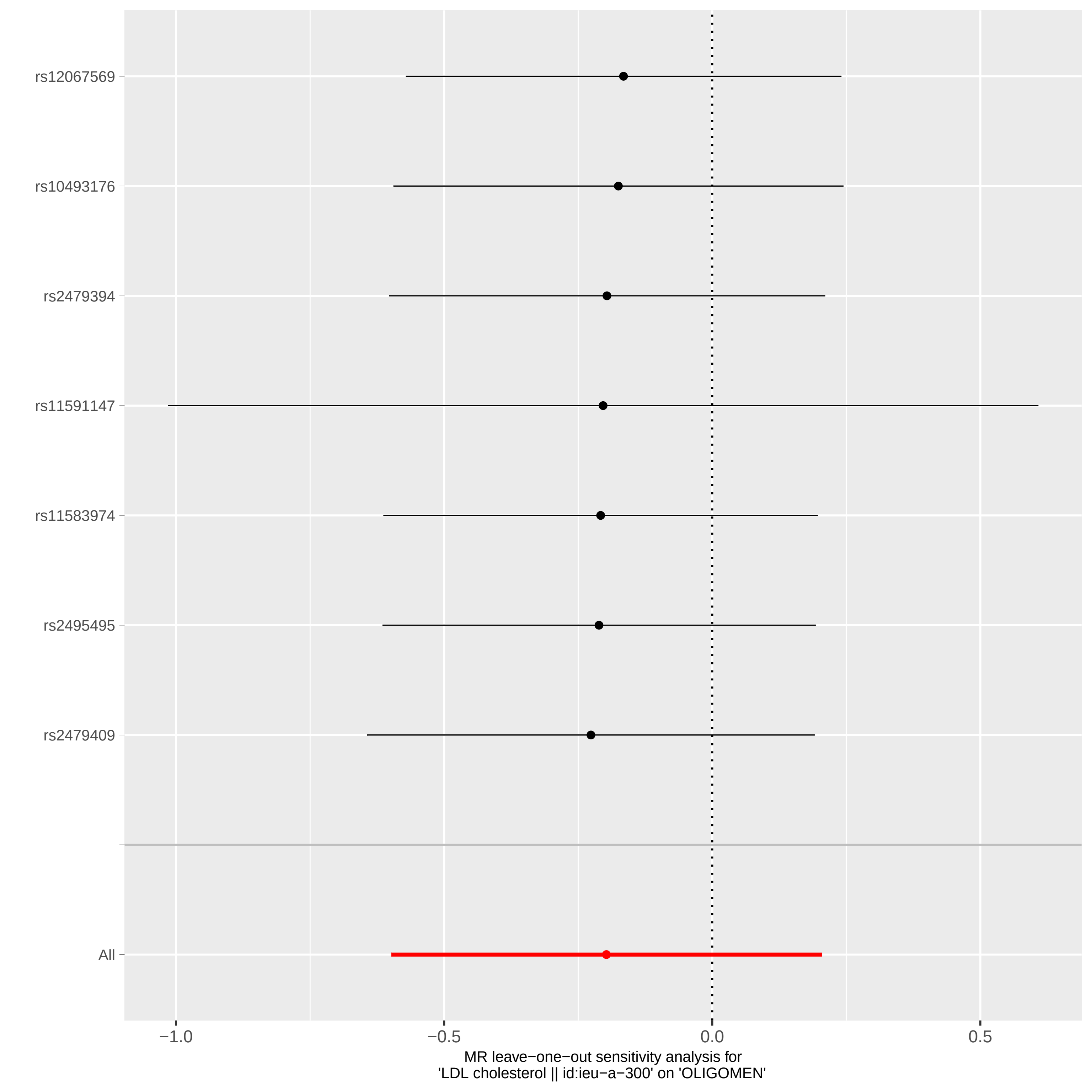
rs12067569
rs10493176
rs2479394
rs11591147
rs11583974
rs2495495
rs2479409
All
−1.0
−0.5
0.0
0.5
MR leave−one−out sensitivity analysis for
'LDL cholesterol || id:ieu−a−300' on 'OLIGOMEN'

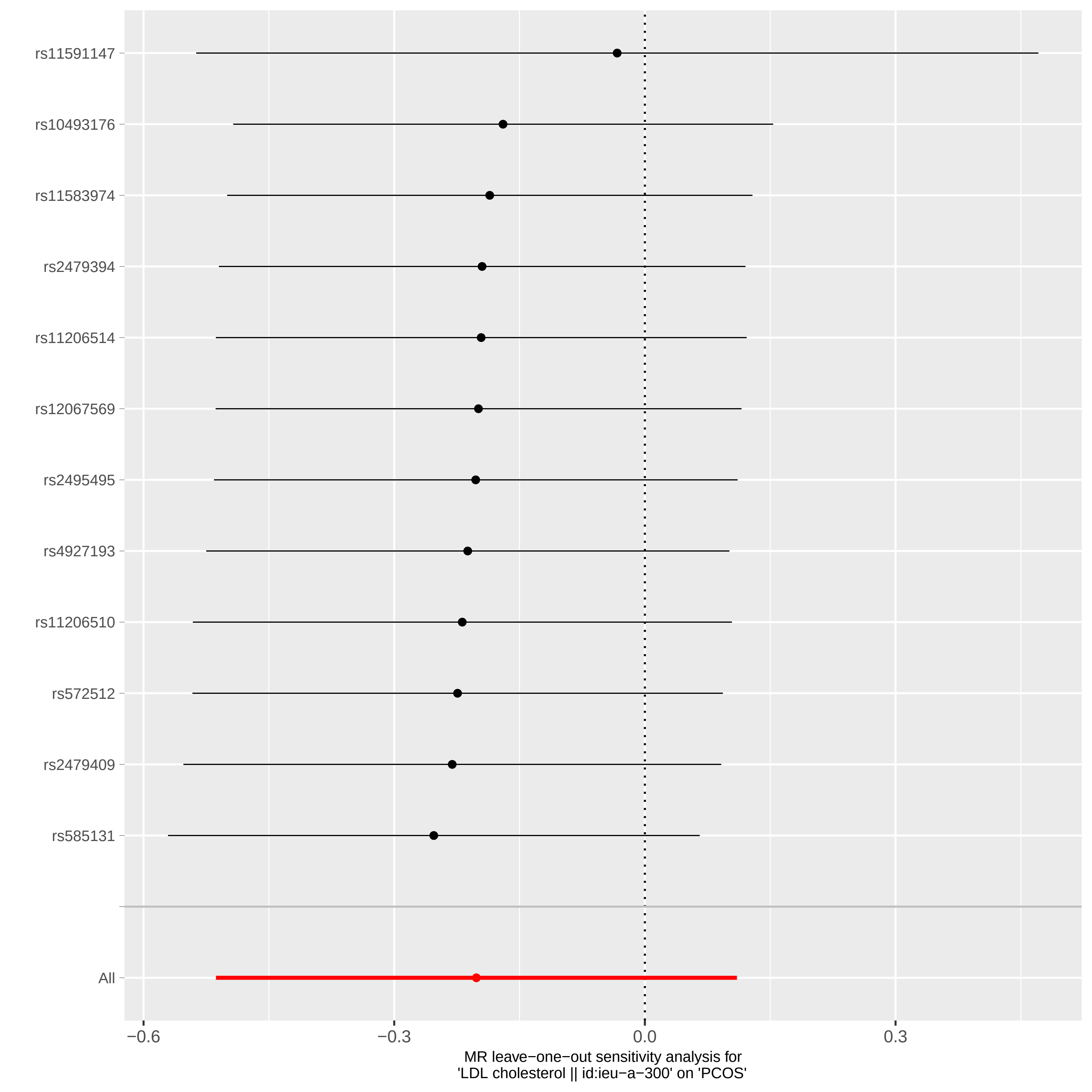
rs11591147
rs10493176
rs11583974
rs2479394
rs11206514
rs12067569
rs2495495
rs4927193
rs11206510
rs572512
rs2479409
rs585131
All
−0.6
−0.3
0.0
0.3
MR leave−one−out sensitivity analysis for
'LDL cholesterol || id:ieu−a−300' on 'PCOS'

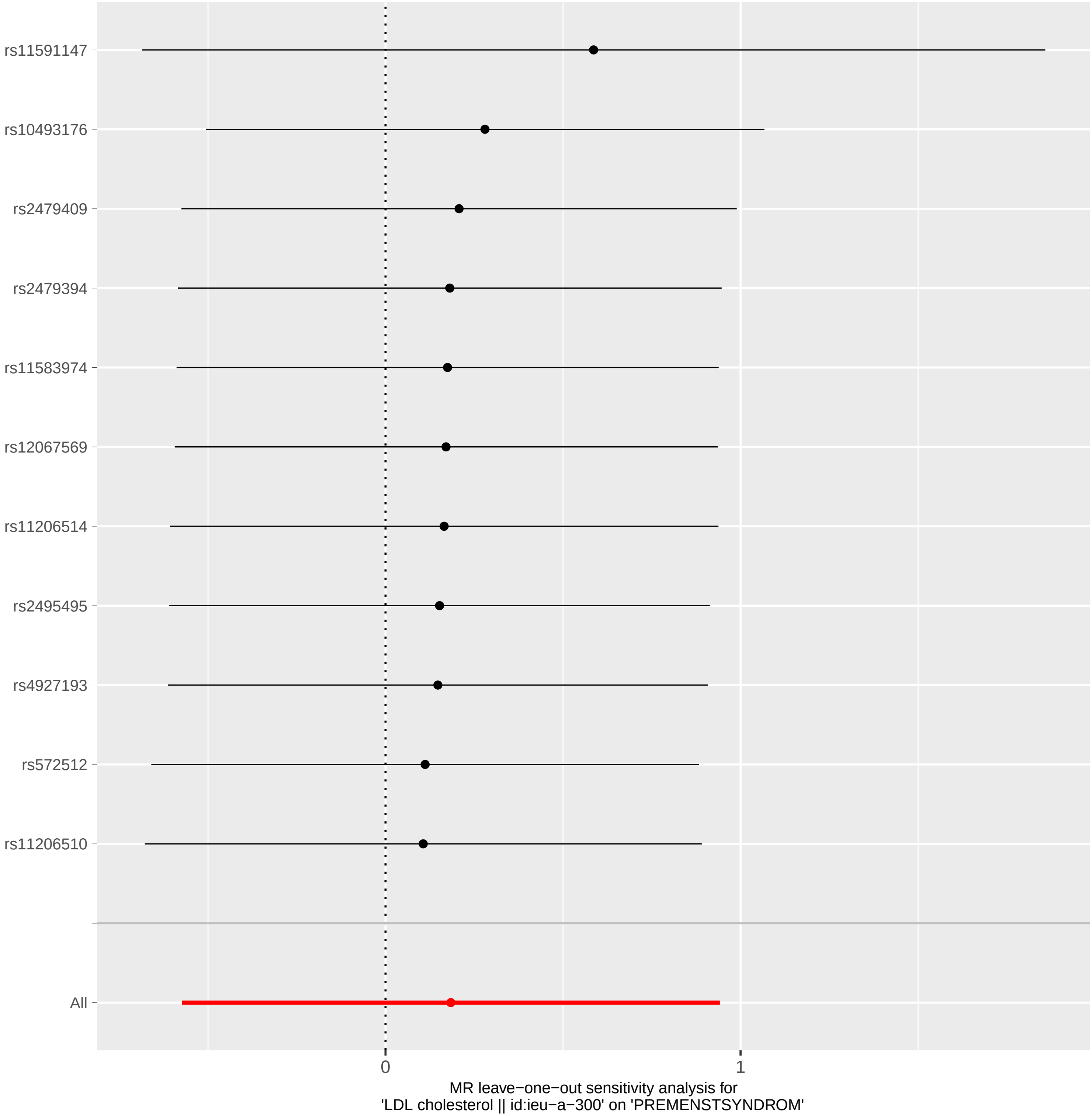
rs11591147
rs10493176
rs2479409
rs2479394
rs11583974
rs12067569
rs11206514
rs2495495
rs4927193
rs572512
rs11206510
All
0
1
MR leave−one−out sensitivity analysis for
'LDL cholesterol || id:ieu−a−300' on 'PREMENSTSYNDROM'

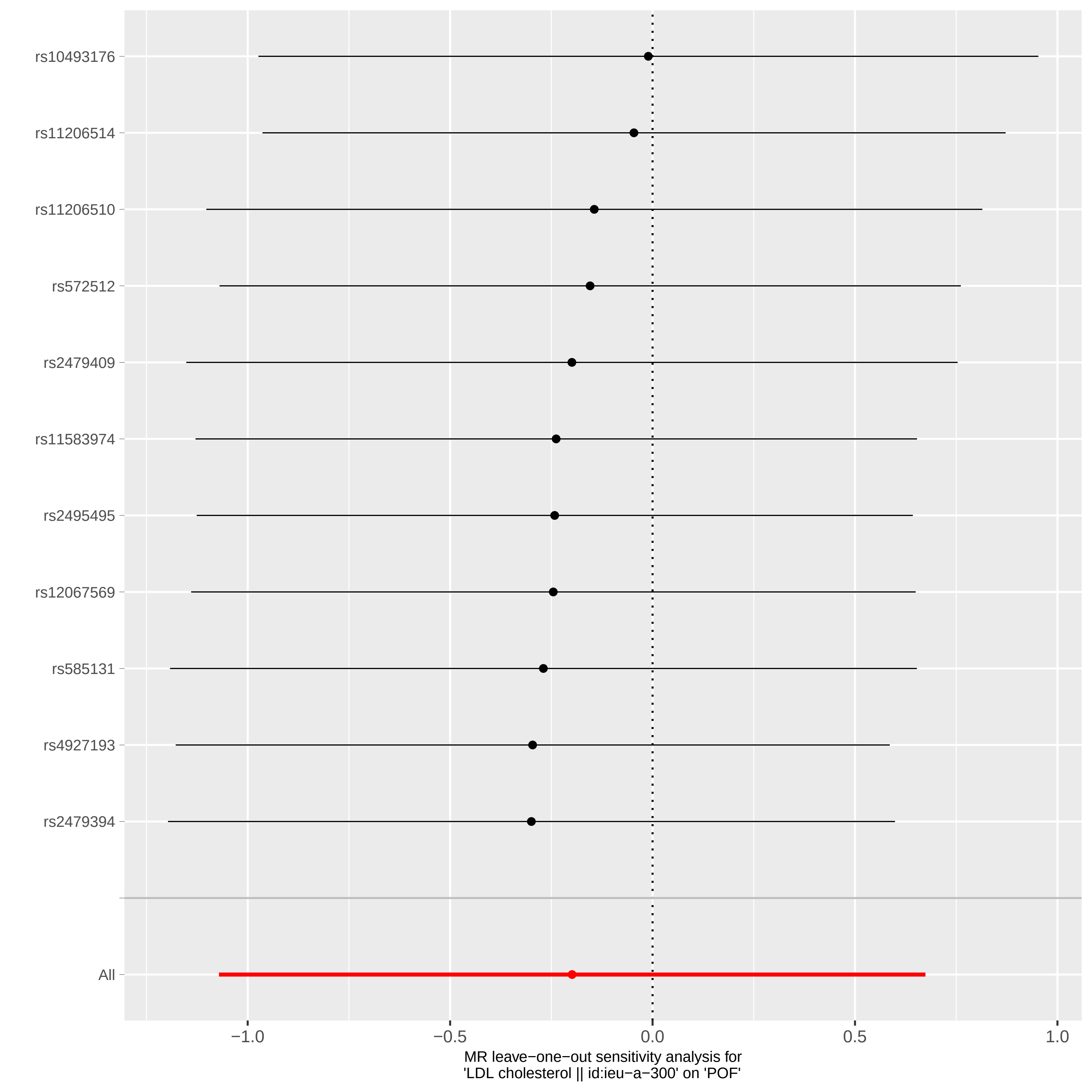
rs10493176
rs11206514
rs11206510
rs572512
rs2479409
rs11583974
rs2495495
rs12067569
rs585131
rs4927193
rs2479394
All
−1.0
−0.5
0.0
0.5
1.0
MR leave−one−out sensitivity analysis for
'LDL cholesterol || id:ieu−a−300' on 'POF'
