# Supplementary material for: Causal association between lipid-lowering drugs and female reproductive endocrine diseases: a drug-targeted Mendelian randomization study
Source: Front Endocrinol (Lausanne). 2023 Nov 10;14:1295412. doi: 10.3389/fendo.2023.1295412 (PMC10668027; doi:10.3389/fendo.2023.1295412)
Supplement: Supplementary file 3 [file DataSheet_2.pdf]

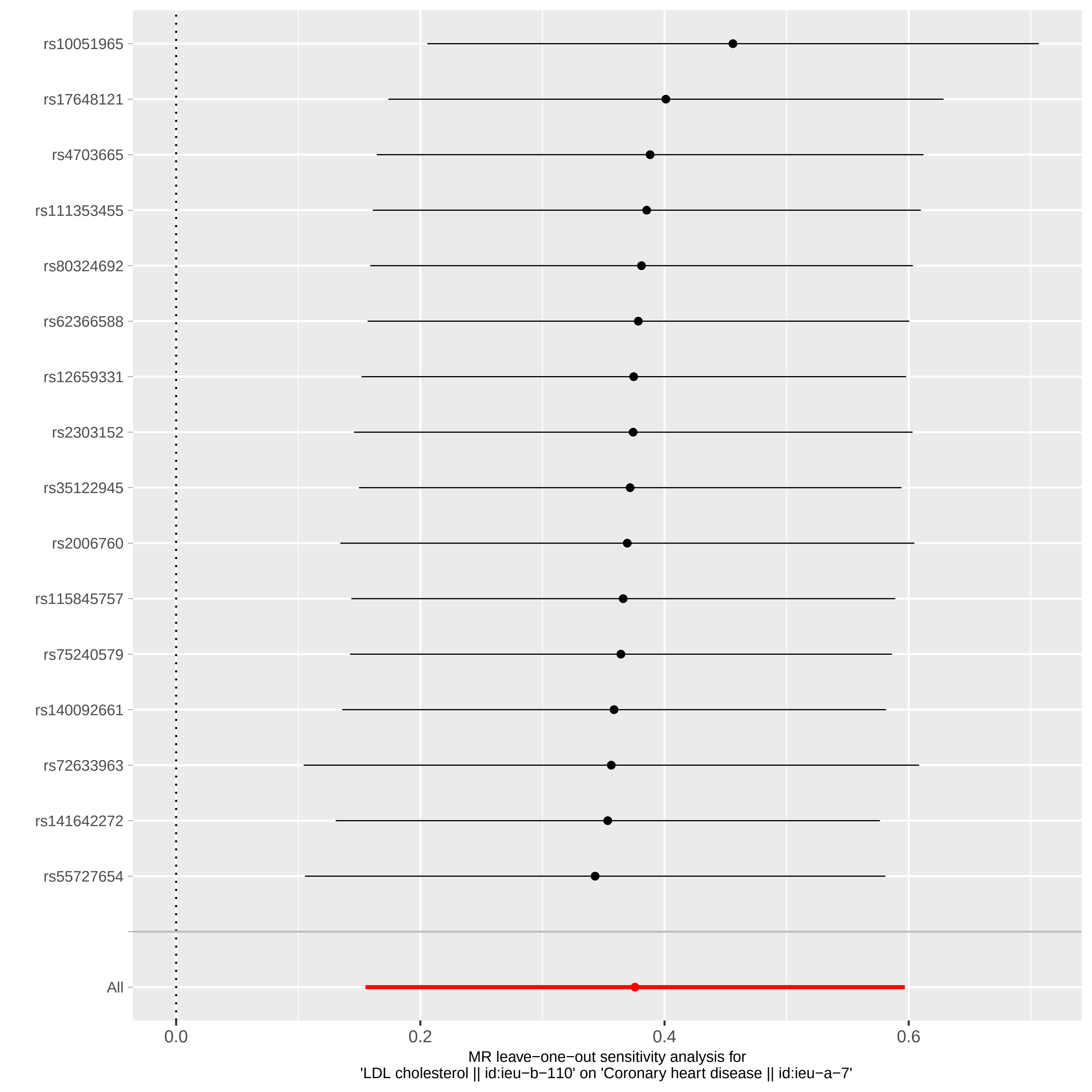
rs10051965
rs17648121
rs4703665
rs111353455
rs80324692
rs62366588
rs12659331
rs2303152
rs35122945
rs2006760
rs115845757
rs75240579
rs140092661
rs72633963
rs141642272
rs55727654
All
0.0
0.2
0.4
0.6
MR leave−one−out sensitivity analysis for
'LDL cholesterol || id:ieu−b−110' on 'Coronary heart disease || id:ieu−a−7'

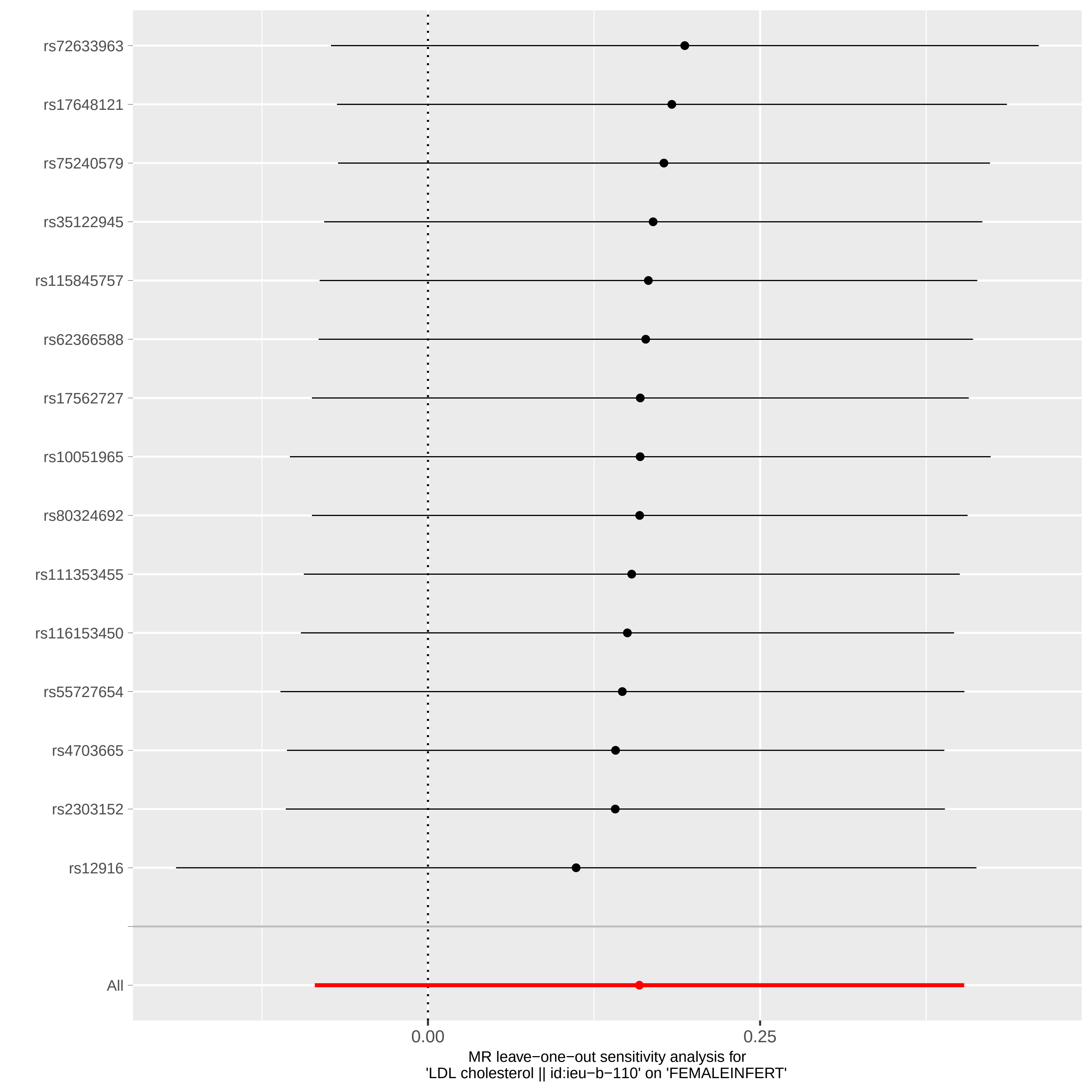
rs72633963
rs17648121
rs75240579
rs35122945
rs115845757
rs62366588
rs17562727
rs10051965
rs80324692
rs111353455
rs116153450
rs55727654
rs4703665
rs2303152
rs12916
All
0.00
0.25
MR leave−one−out sensitivity analysis for
'LDL cholesterol || id:ieu−b−110' on 'FEMALEINFERT'

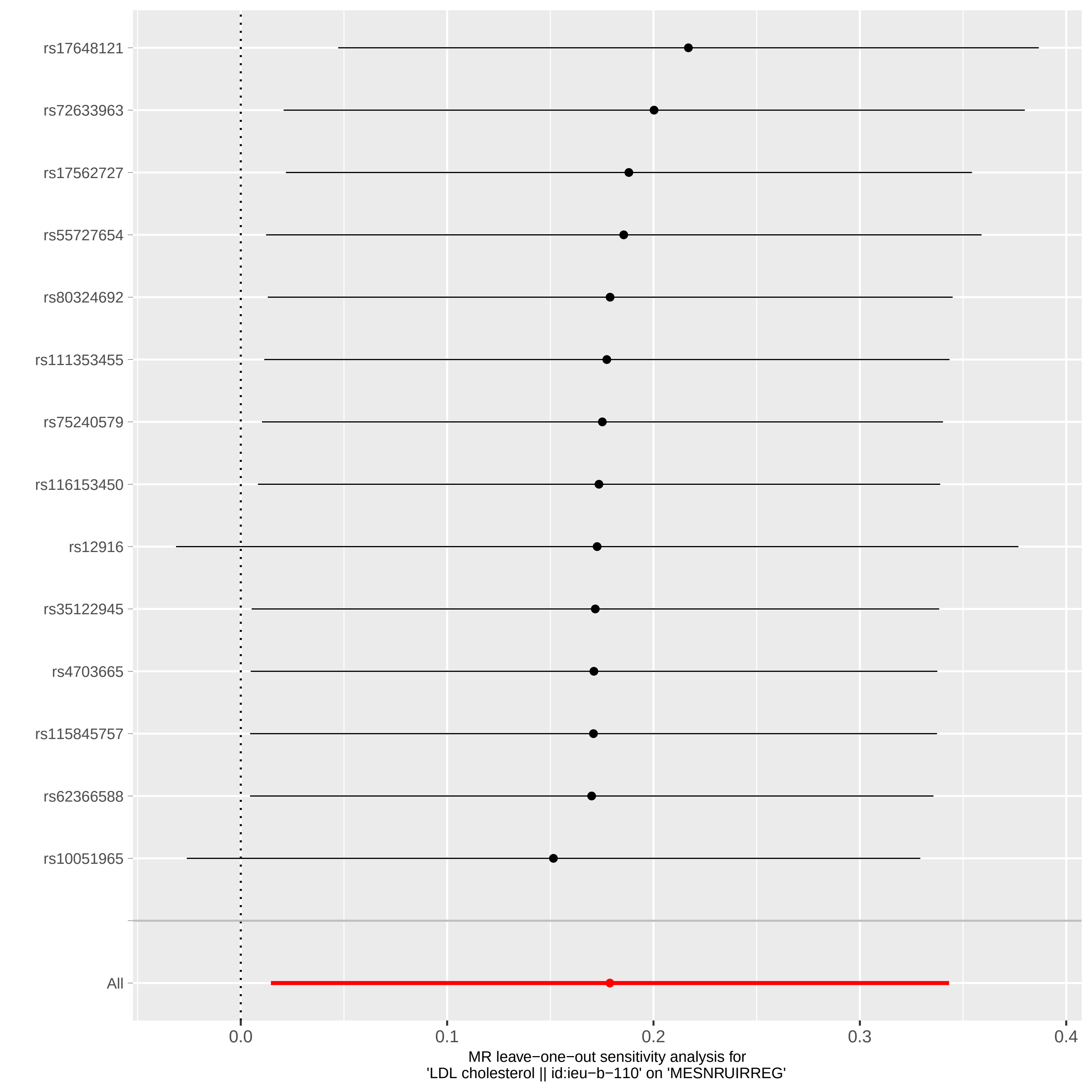
rs17648121
rs72633963
rs17562727
rs55727654
rs80324692
rs111353455
rs75240579
rs116153450
rs12916
rs35122945
rs4703665
rs115845757
rs62366588
rs10051965
All
0.0
0.1
0.2
0.3
0.4
MR leave−one−out sensitivity analysis for
'LDL cholesterol || id:ieu−b−110' on 'MESNRUIRREG'

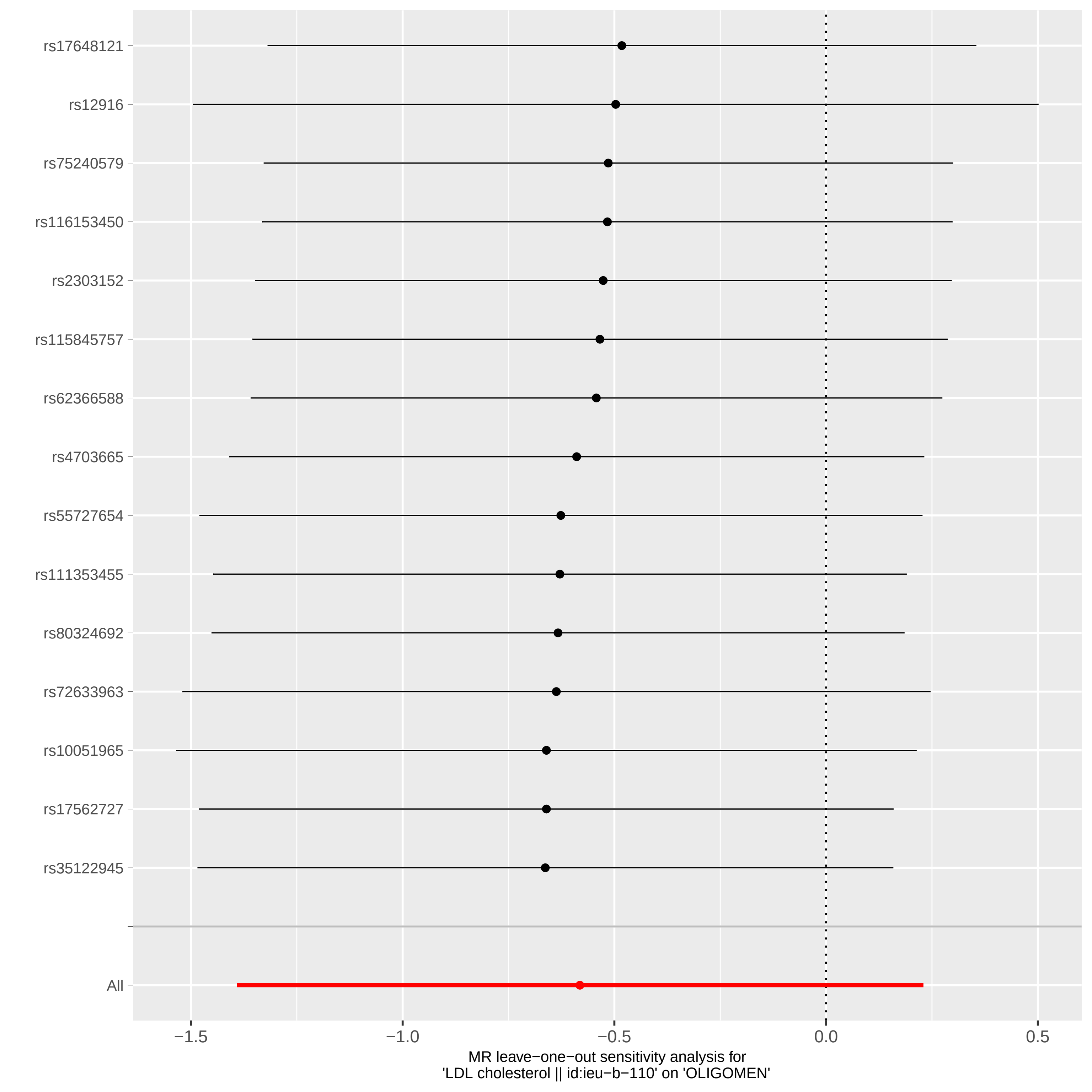
rs17648121
rs12916
rs75240579
rs116153450
rs2303152
rs115845757
rs62366588
rs4703665
rs55727654
rs111353455
rs80324692
rs72633963
rs10051965
rs17562727
rs35122945
All
−1.5
−1.0
−0.5
0.0
0.5
MR leave−one−out sensitivity analysis for
'LDL cholesterol || id:ieu−b−110' on 'OLIGOMEN'

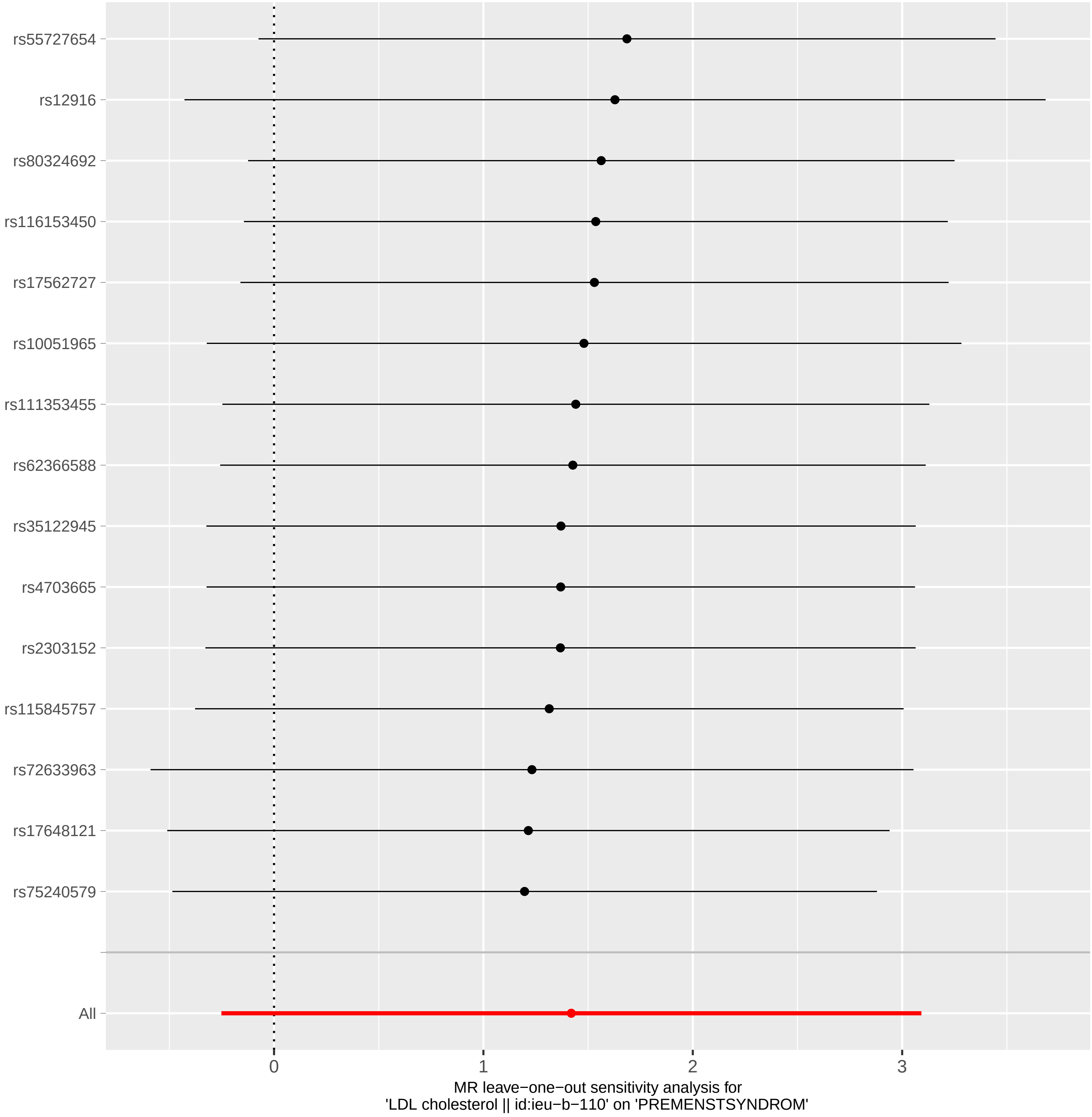

rs55727654
rs12916
rs80324692
rs116153450
rs17562727
rs10051965
rs111353455
rs62366588
rs35122945
rs4703665
rs2303152
rs115845757
rs72633963
rs17648121
rs75240579
All
0
1
2
3
MR leave−one−out sensitivity analysis for
'LDL cholesterol || id:ieu−b−110' on 'PREMENSTSYNDROM'

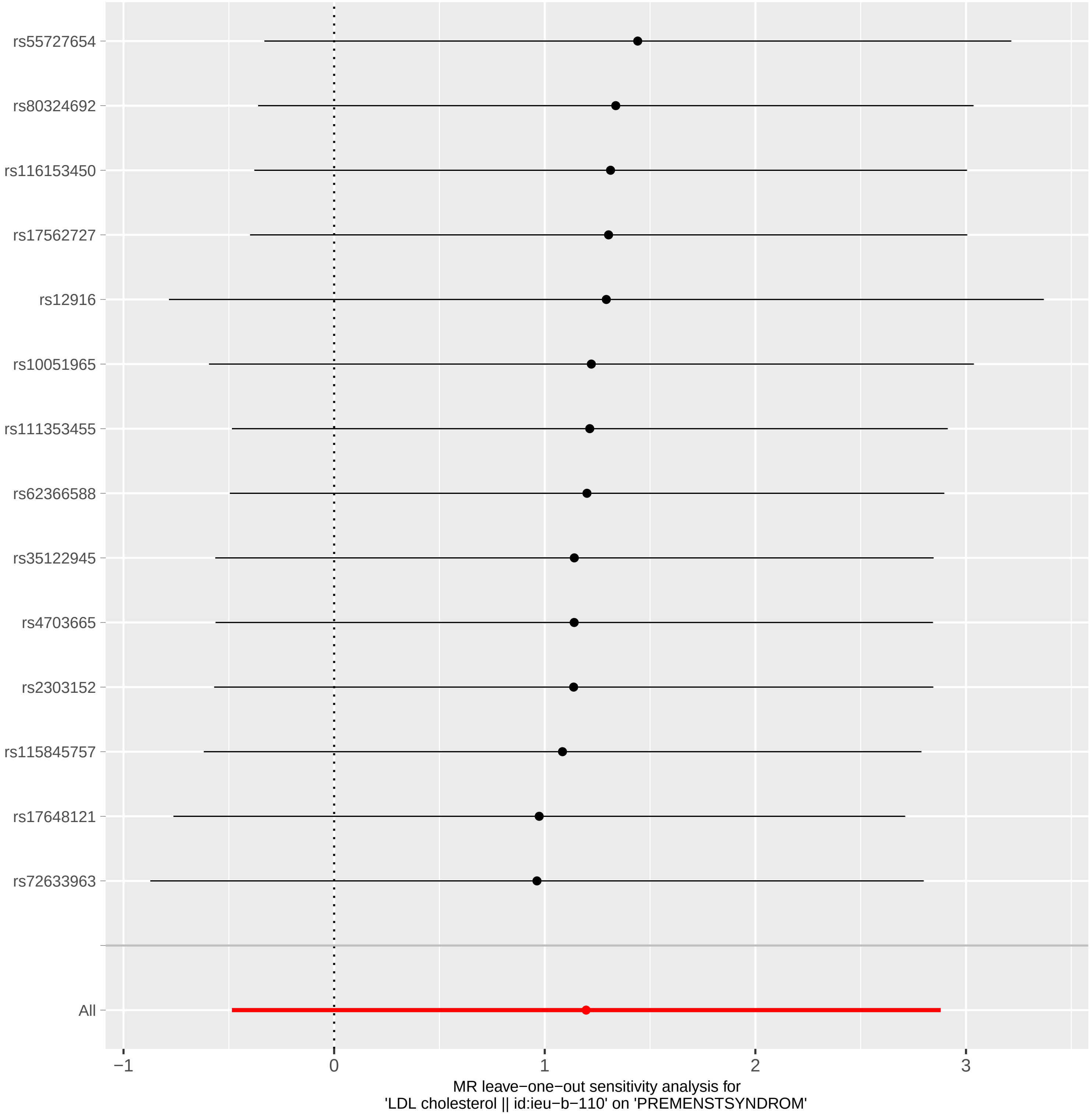
rs55727654
rs80324692
rs116153450
rs17562727
rs12916
rs10051965
rs111353455
rs62366588
rs35122945
rs4703665
rs2303152
rs115845757
rs17648121
rs72633963
All
−1
0
1
2
3
MR leave−one−out sensitivity analysis for
'LDL cholesterol || id:ieu−b−110' on 'PREMENSTSYNDROM'

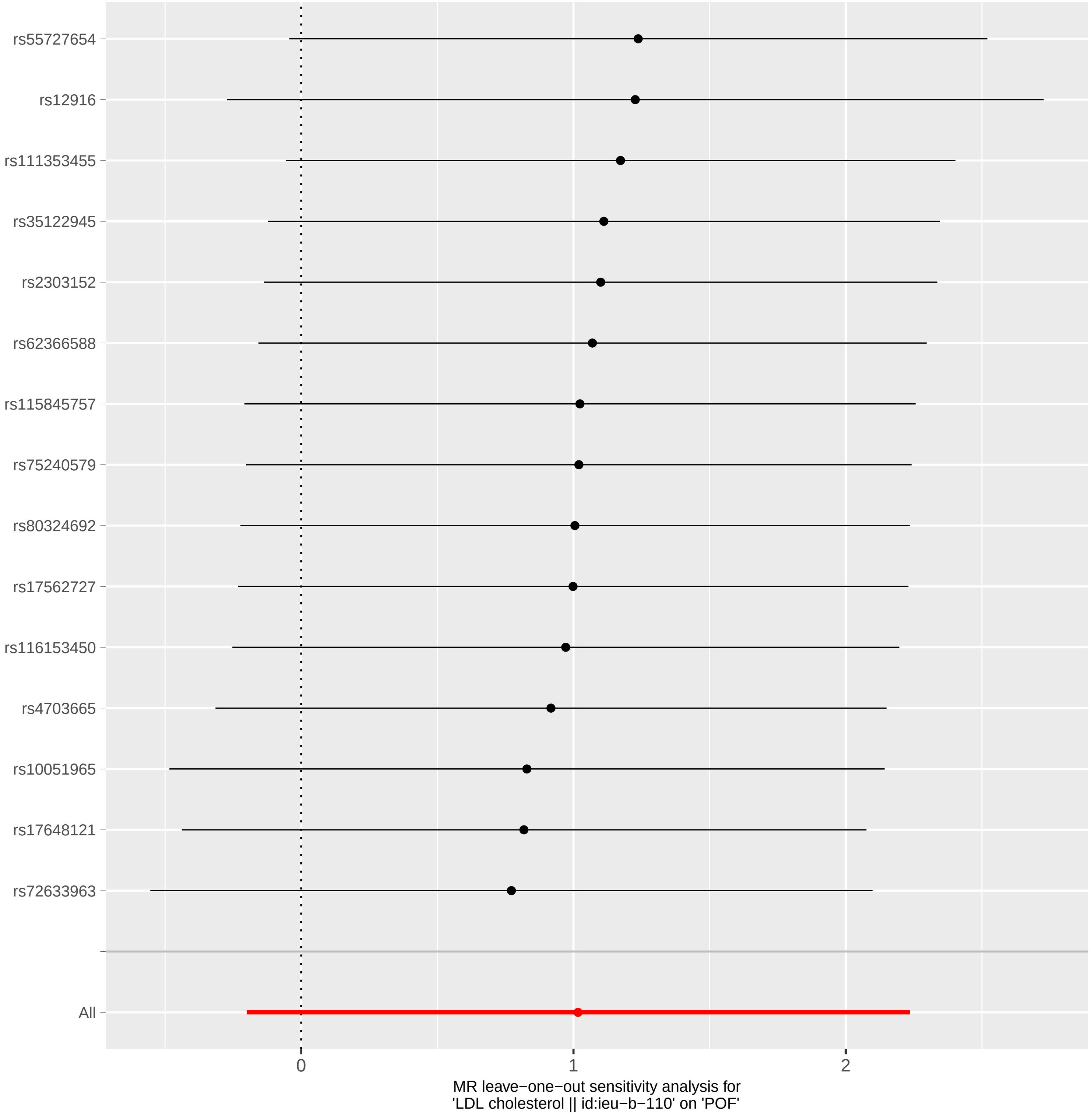
rs55727654
rs12916
rs111353455
rs35122945
rs2303152
rs62366588
rs115845757
rs75240579
rs80324692
rs17562727
rs116153450
rs4703665
rs10051965
rs17648121
rs72633963
All
0
1
2
MR leave−one−out sensitivity analysis for
'LDL cholesterol || id:ieu−b−110' on 'POF'

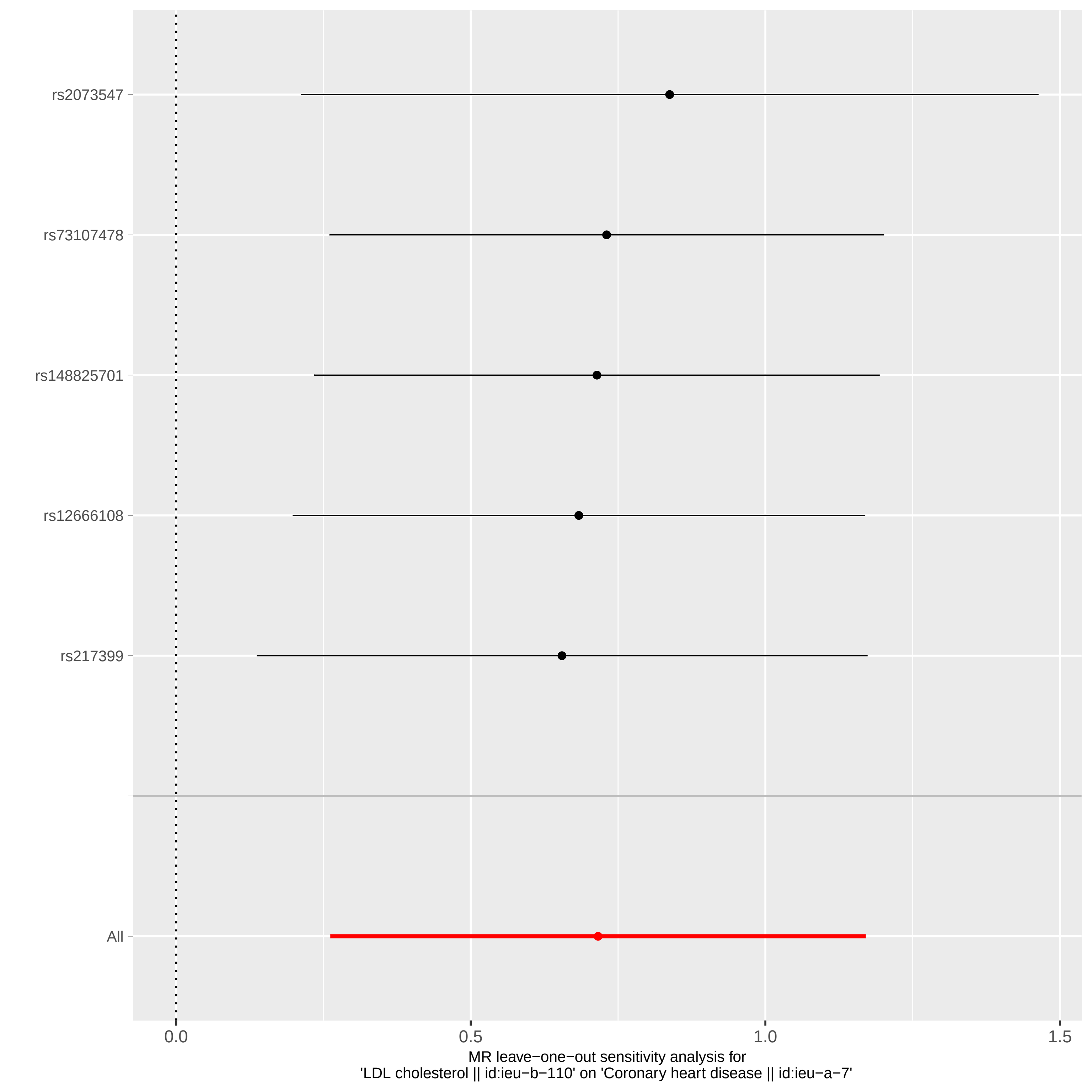

rs2073547
rs73107478
rs148825701
rs12666108
rs217399
All
0.0
0.5
1.0
1.5
MR leave−one−out sensitivity analysis for
'LDL cholesterol || id:ieu−b−110' on 'Coronary heart disease || id:ieu−a−7'

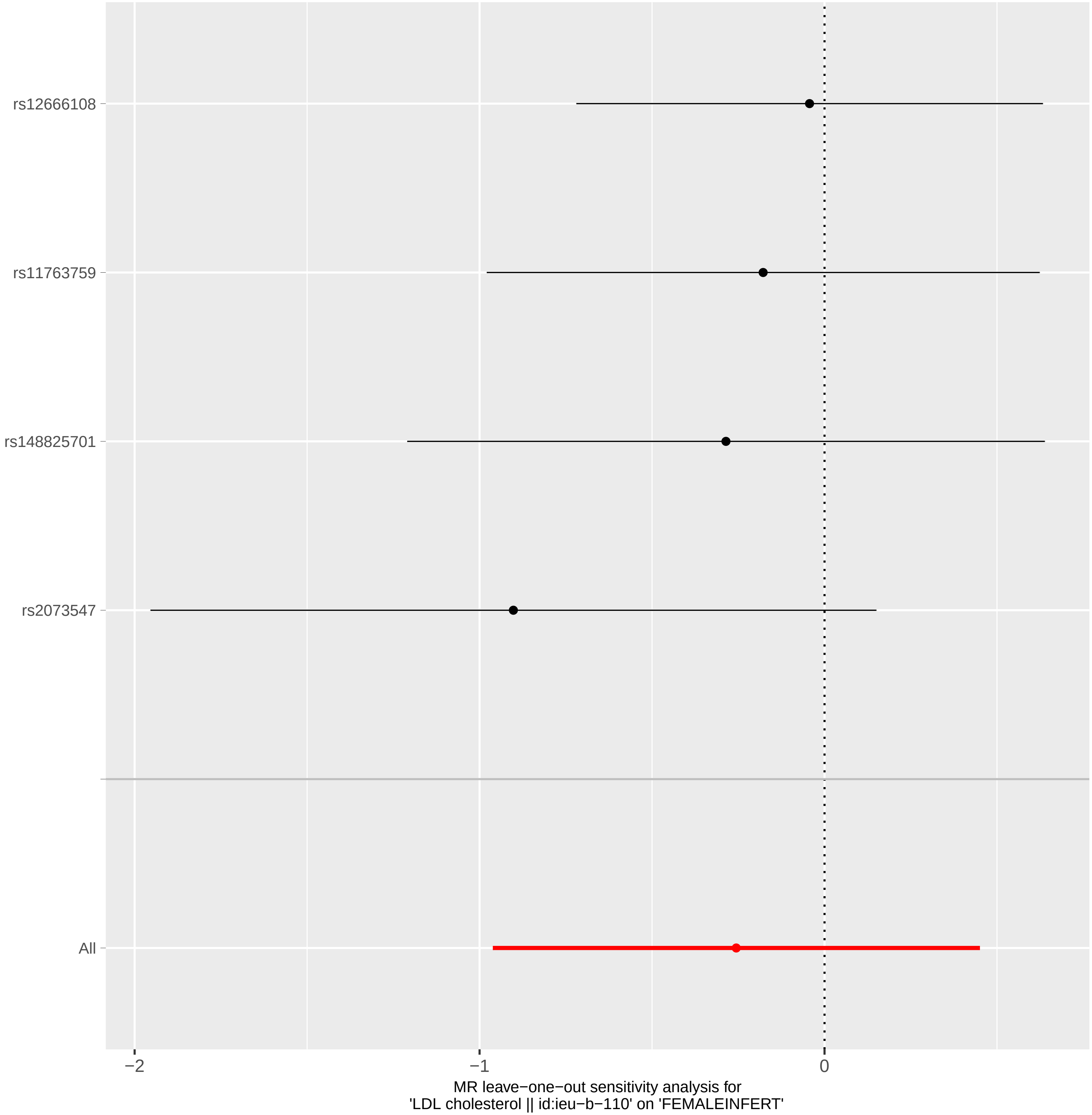
rs12666108
rs11763759
rs148825701
rs2073547
All
−2
−1
0
MR leave−one−out sensitivity analysis for
'LDL cholesterol || id:ieu−b−110' on 'FEMALEINFERT'

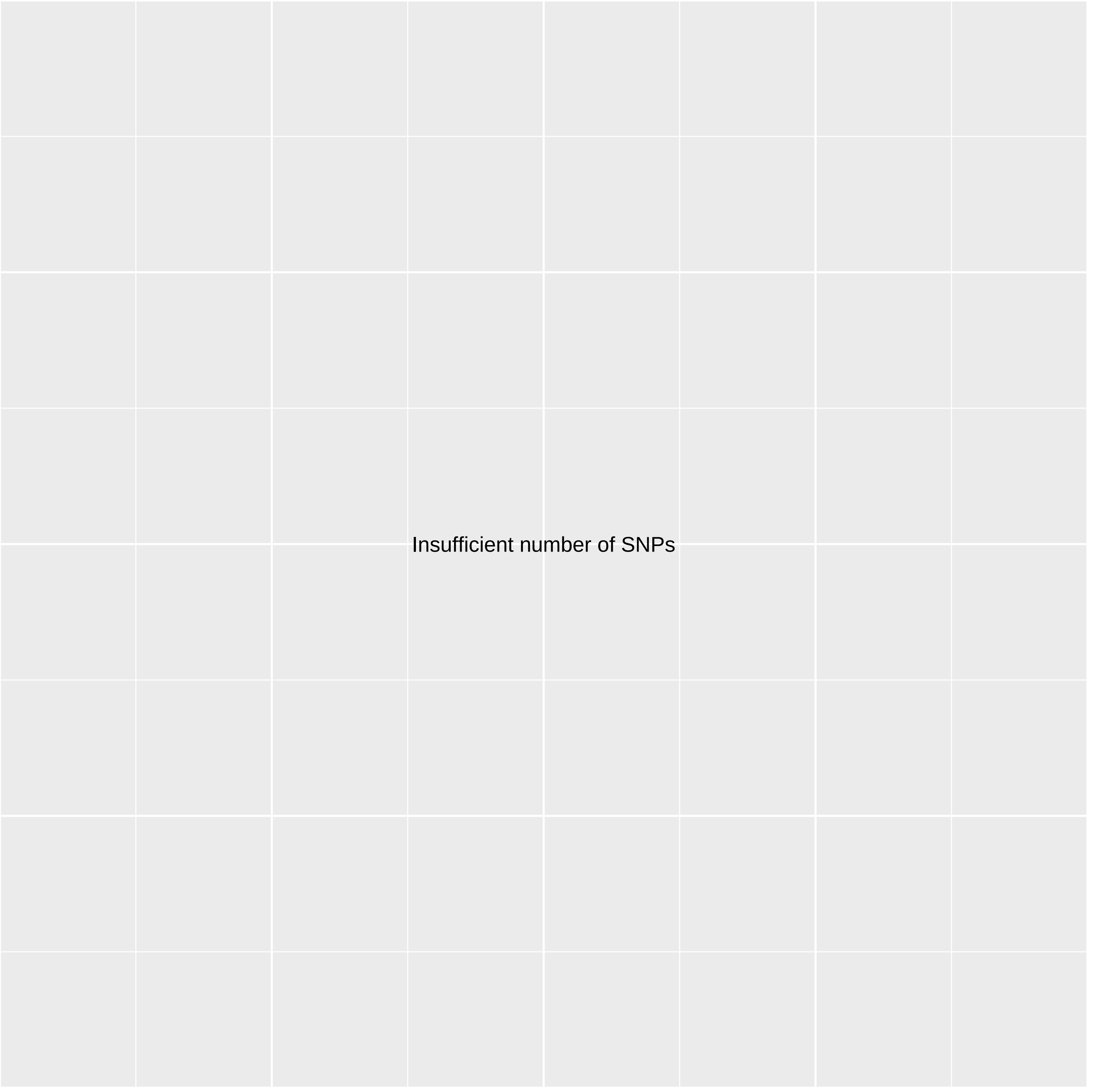
Insufficient number of SNPs

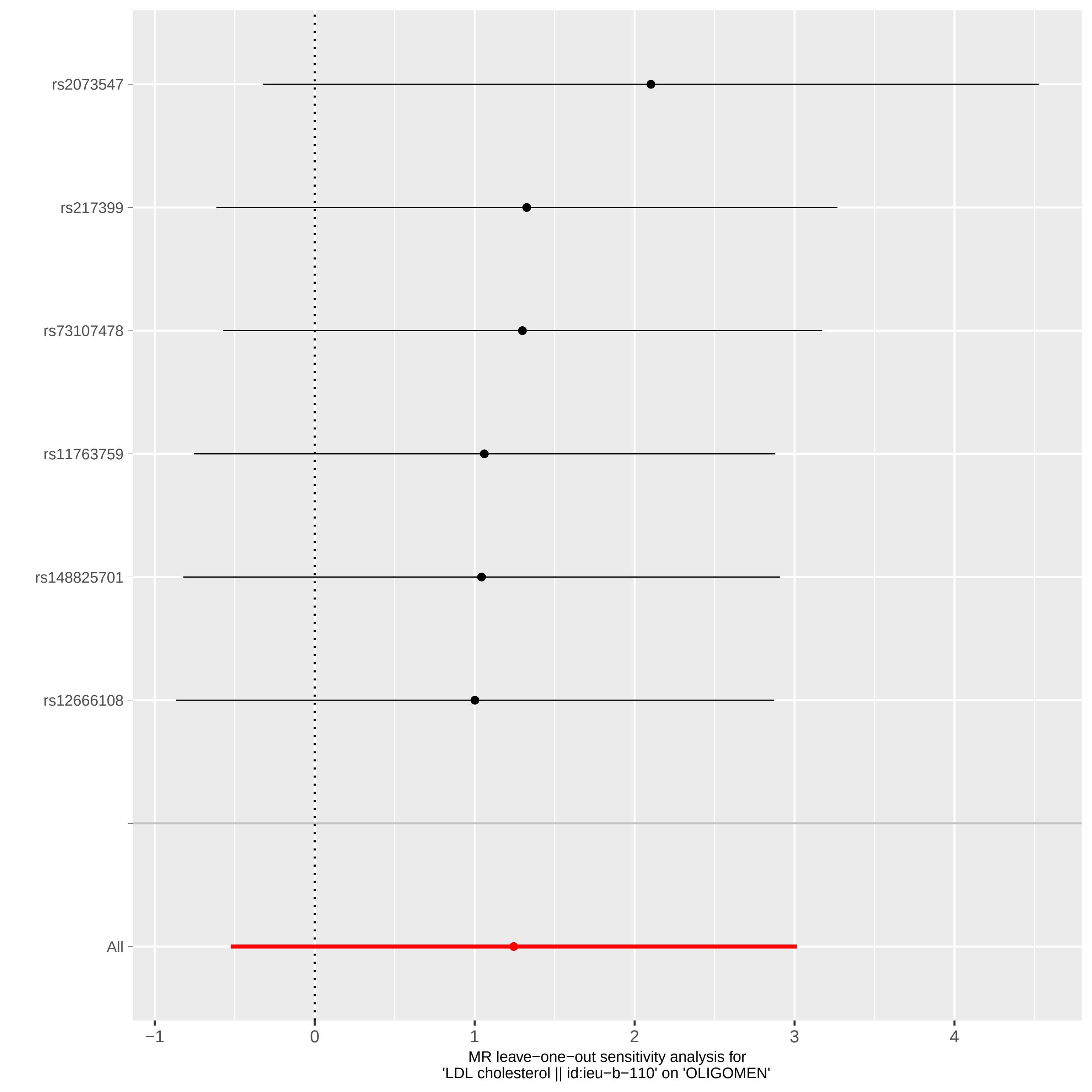
rs2073547
rs217399
rs73107478
rs11763759
rs148825701
rs12666108
All
−1
0
1
2
3
4
MR leave−one−out sensitivity analysis for
'LDL cholesterol || id:ieu−b−110' on 'OLIGOMEN'

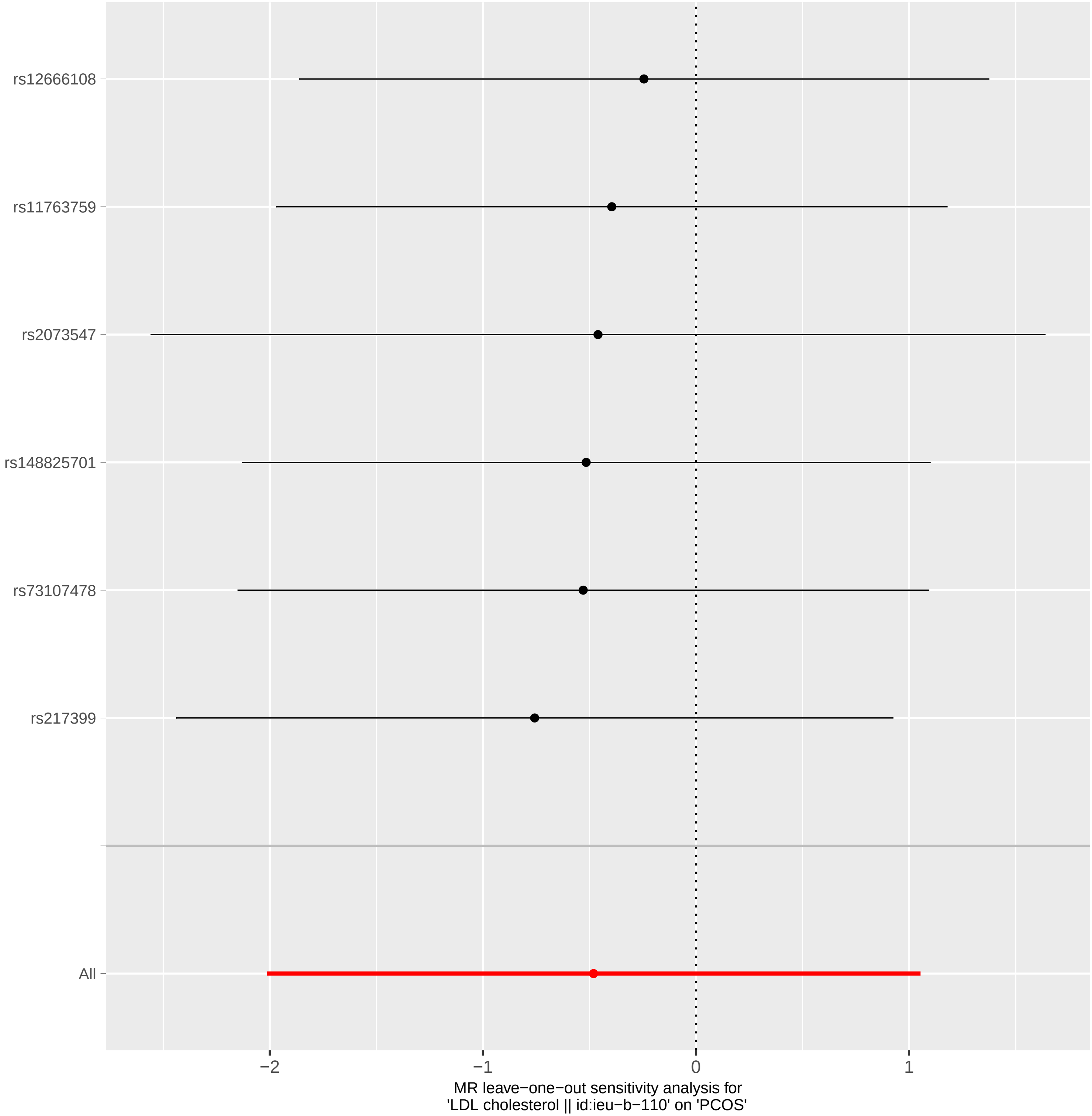
rs12666108
rs11763759
rs2073547
rs148825701
rs73107478
rs217399
All
−2
−1
0
1
MR leave−one−out sensitivity analysis for
'LDL cholesterol || id:ieu−b−110' on 'PCOS'

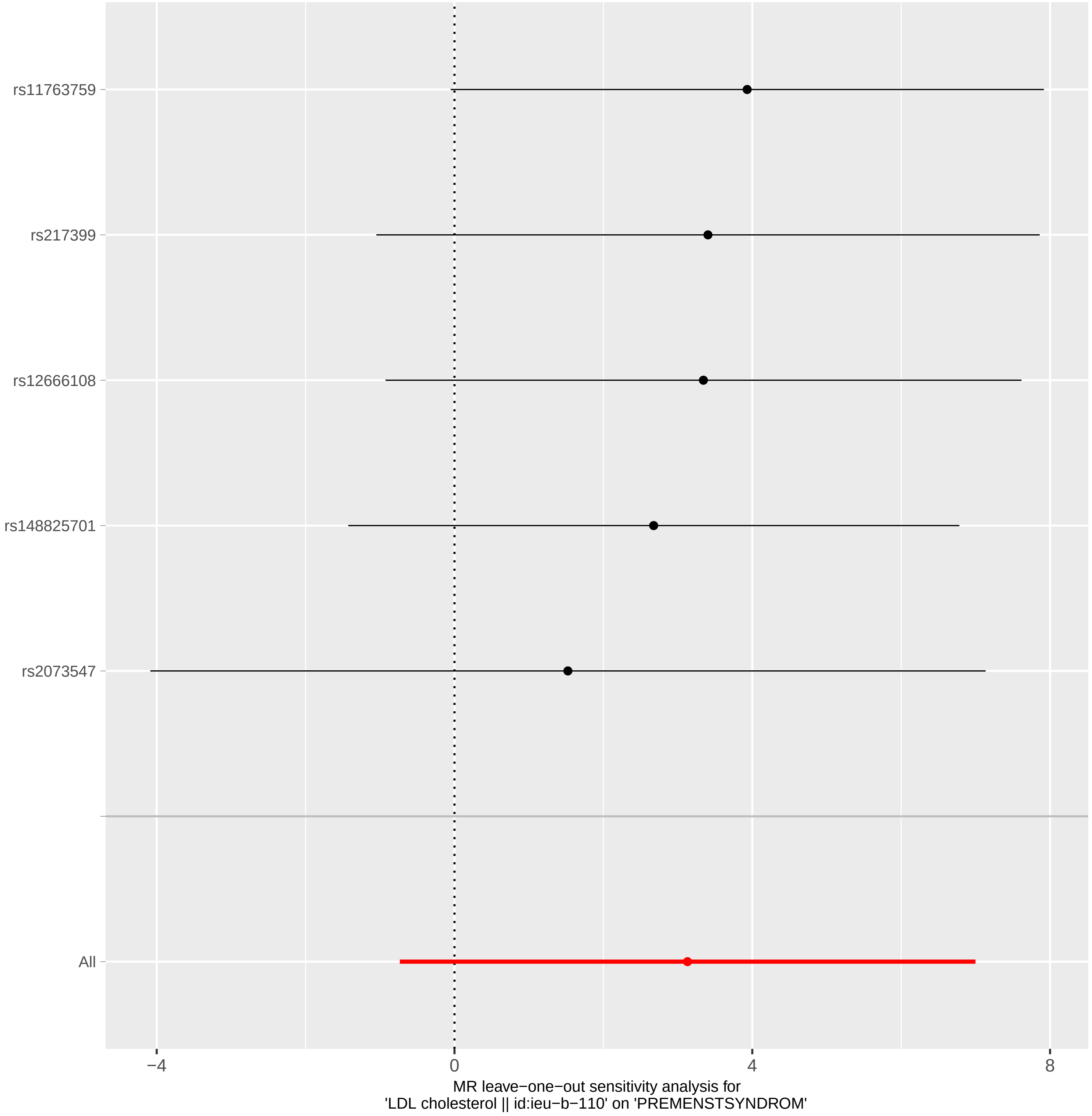
rs11763759
rs217399
rs12666108
rs148825701
rs2073547
All
−4
0
4
8
MR leave−one−out sensitivity analysis for
'LDL cholesterol || id:ieu−b−110' on 'PREMENSTSYNDROM'

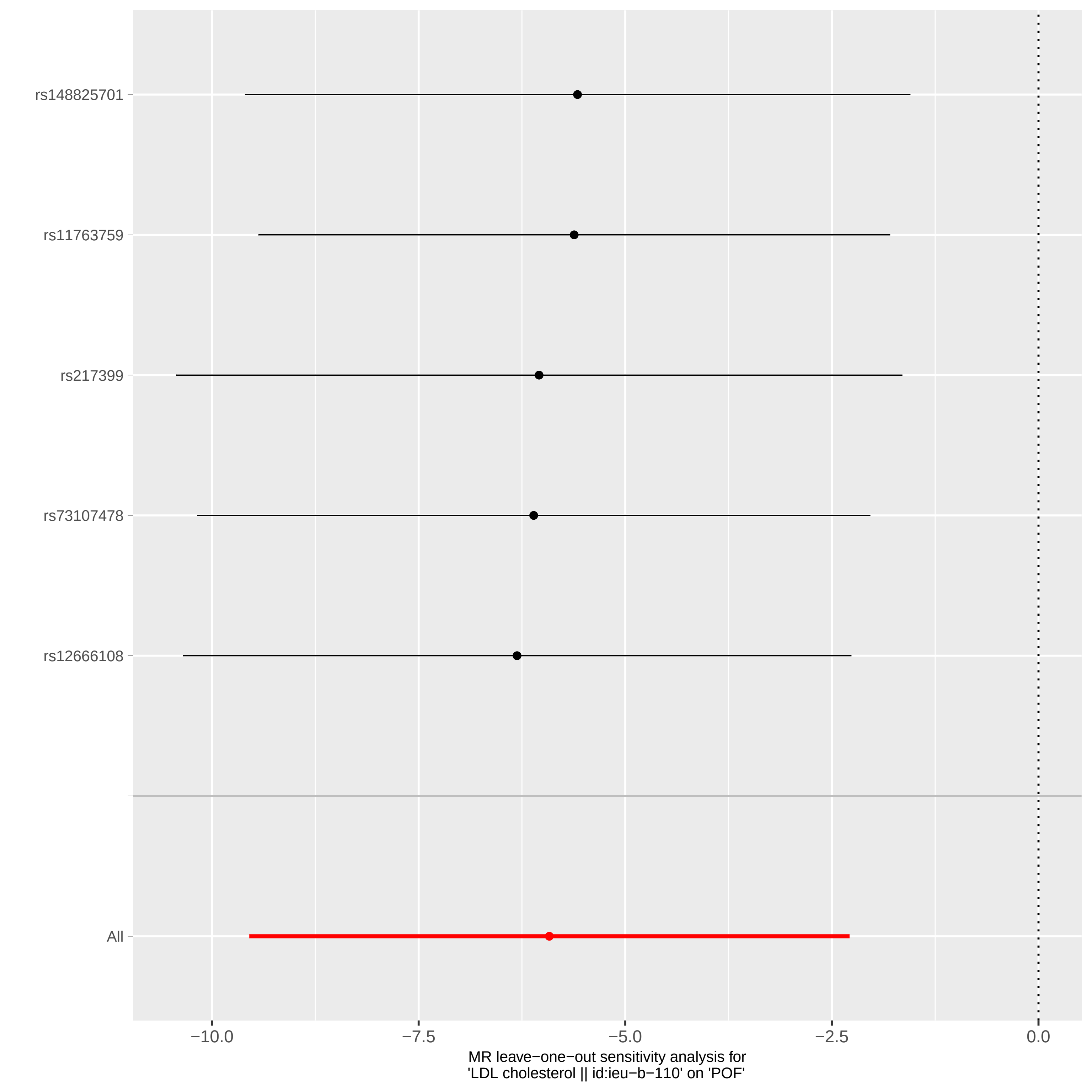
rs148825701
rs11763759
rs217399
rs73107478
rs12666108
All
−10.0
−7.5
−5.0
−2.5
0.0
MR leave−one−out sensitivity analysis for
'LDL cholesterol || id:ieu−b−110' on 'POF'

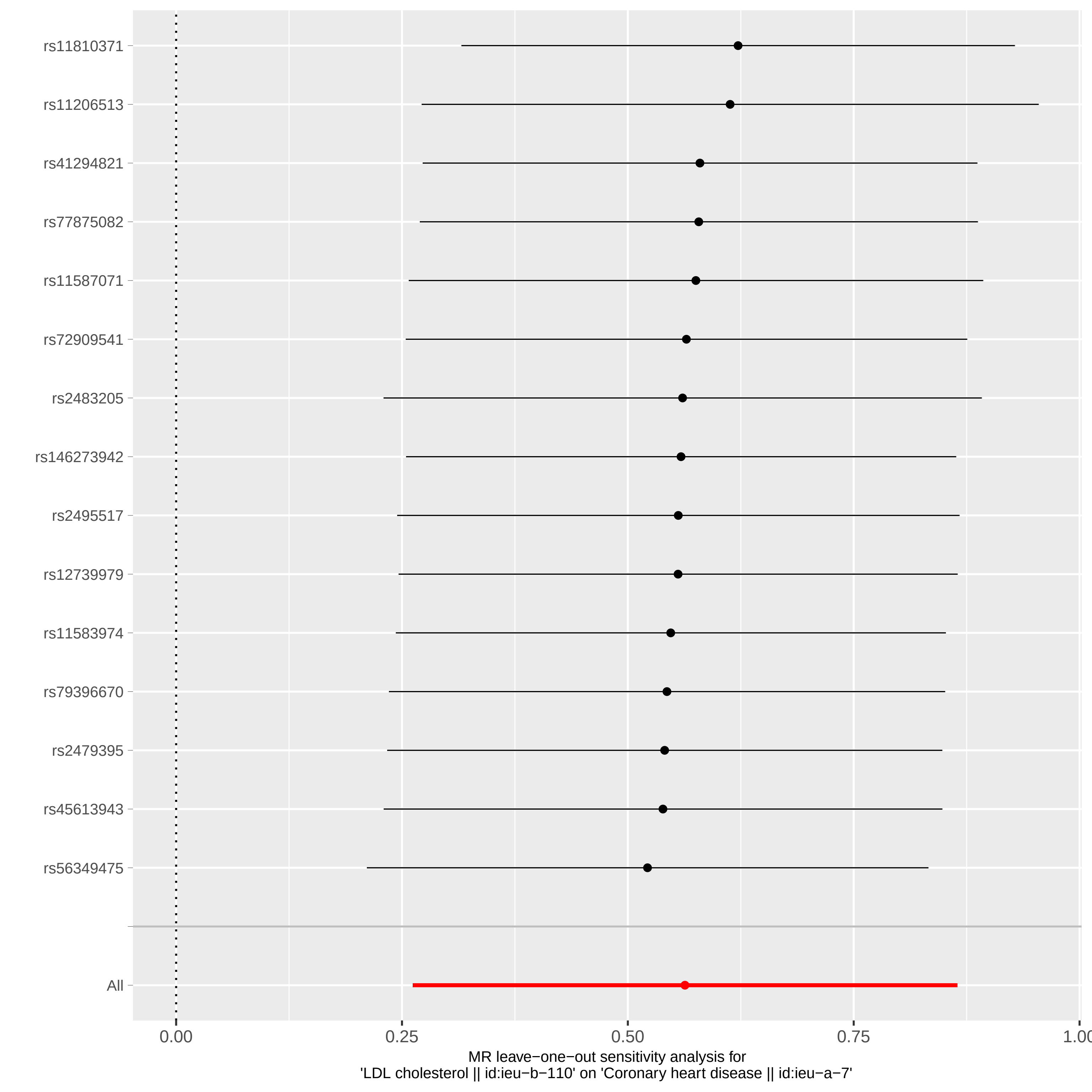
rs11810371
rs11206513
rs41294821
rs77875082
rs11587071
rs72909541
rs2483205
rs146273942
rs2495517
rs12739979
rs11583974
rs79396670
rs2479395
rs45613943
rs56349475
All
0.00
0.25
0.50
0.75
1.00
MR leave−one−out sensitivity analysis for
'LDL cholesterol || id:ieu−b−110' on 'Coronary heart disease || id:ieu−a−7'

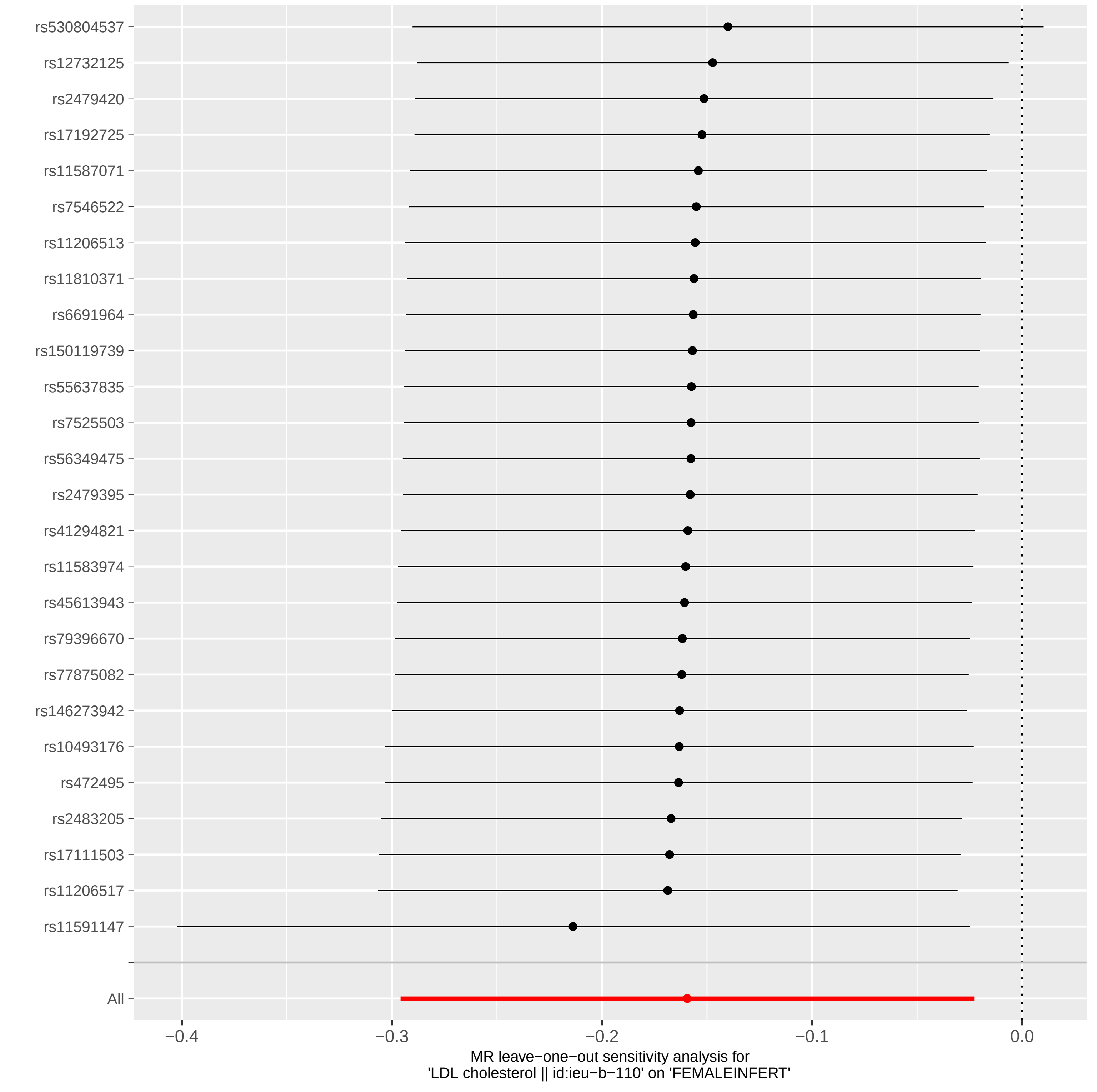
rs530804537
rs12732125
rs2479420
rs17192725
rs11587071
rs7546522
rs11206513
rs11810371
rs6691964
rs150119739
rs55637835
rs7525503
rs56349475
rs2479395
rs41294821
rs11583974
rs45613943
rs79396670
rs77875082
rs146273942
rs10493176
rs472495
rs2483205
rs17111503
rs11206517
rs11591147
All
−0.4
−0.3
−0.2
−0.1
0.0
MR leave−one−out sensitivity analysis for
'LDL cholesterol || id:ieu−b−110' on 'FEMALEINFERT'

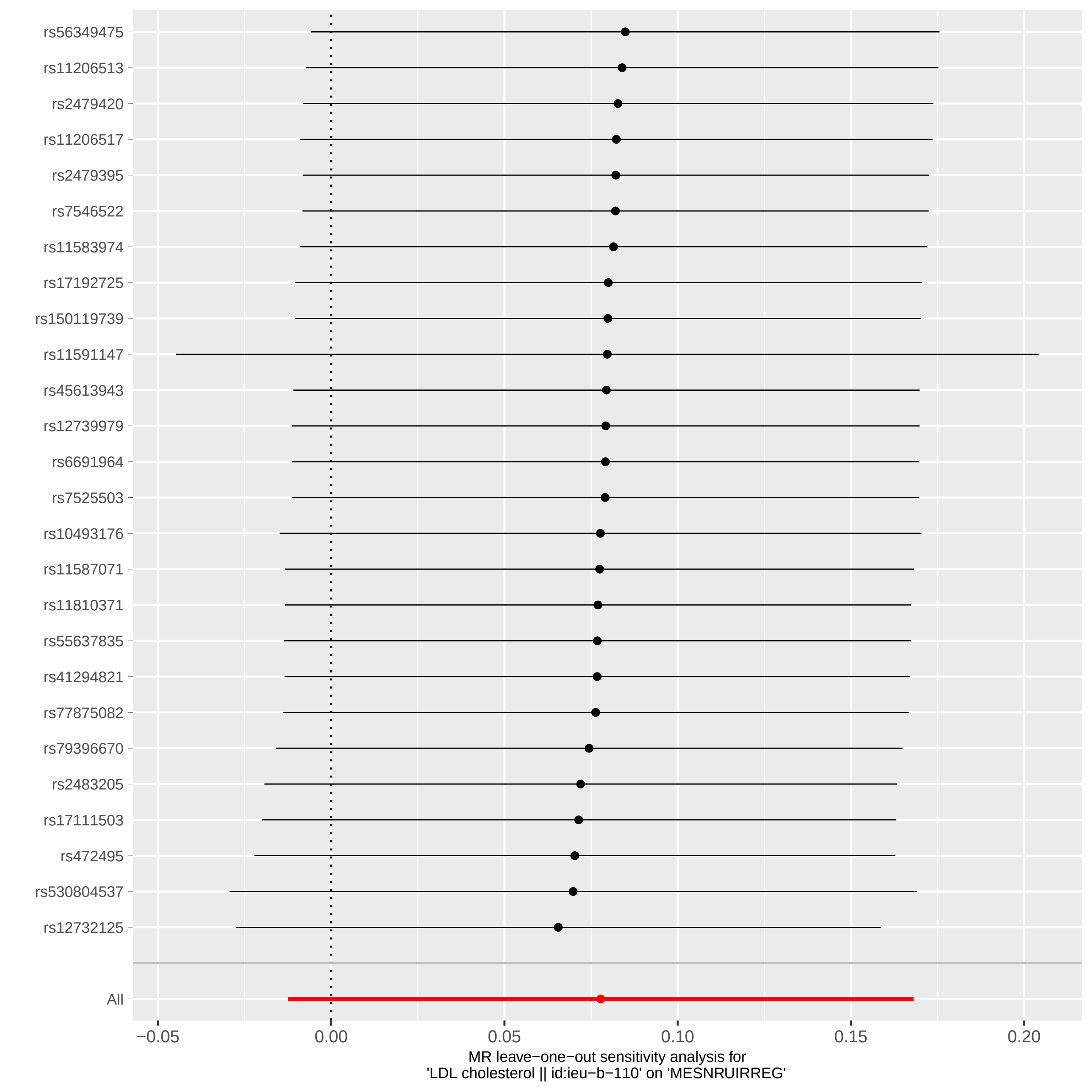

rs56349475
rs11206513
rs2479420
rs11206517
rs2479395
rs7546522
rs11583974
rs17192725
rs150119739
rs11591147
rs45613943
rs12739979
rs6691964
rs7525503
rs10493176
rs11587071
rs11810371
rs55637835
rs41294821
rs77875082
rs79396670
rs2483205
rs17111503
rs472495
rs530804537
rs12732125
All
−0.05
0.00
0.05
0.10
0.15
0.20
MR leave−one−out sensitivity analysis for
'LDL cholesterol || id:ieu−b−110' on 'MESNRUIRREG'

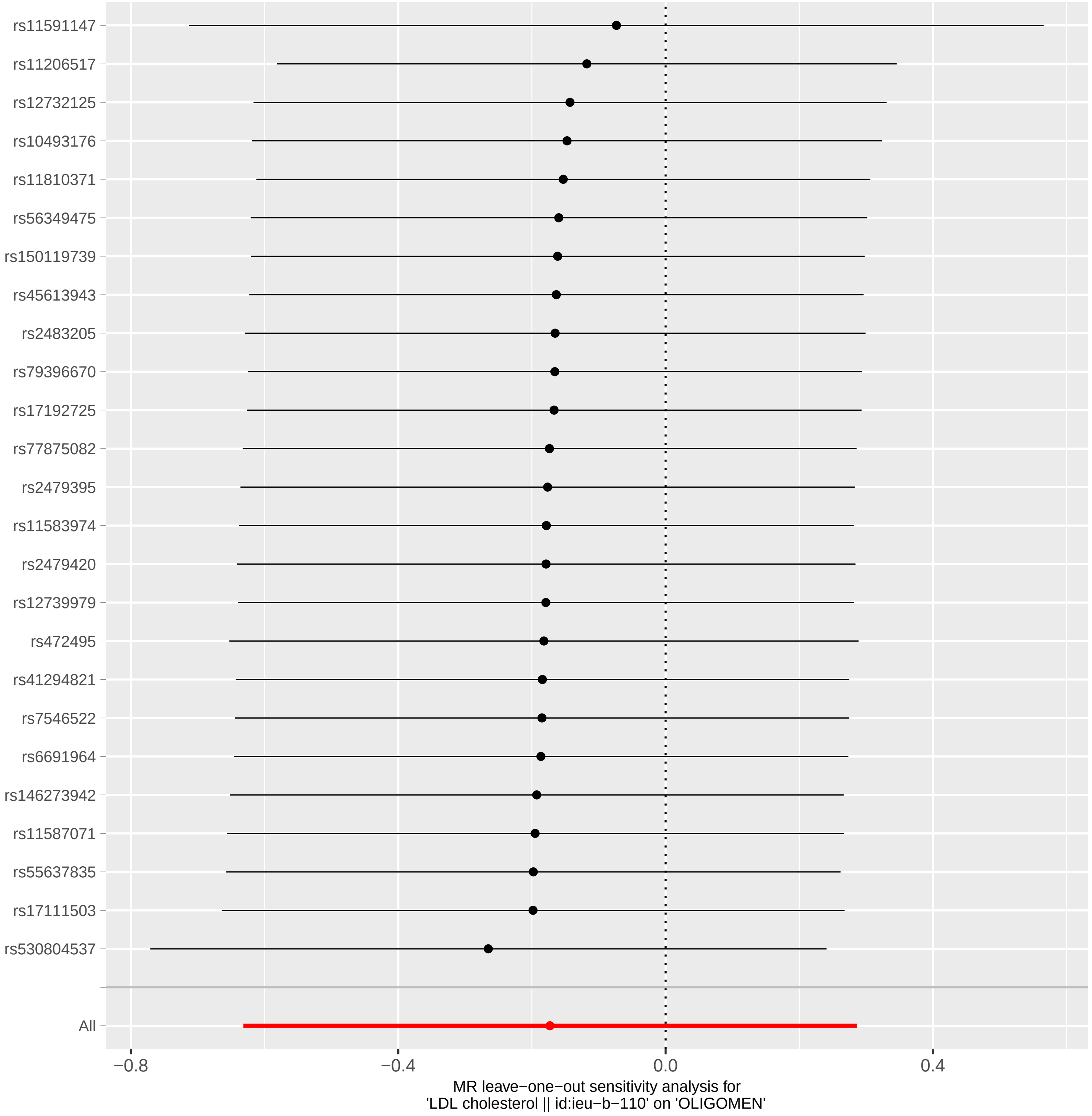
rs11591147
rs11206517
rs12732125
rs10493176
rs11810371
rs56349475
rs150119739
rs45613943
rs2483205
rs79396670
rs17192725
rs77875082
rs2479395
rs11583974
rs2479420
rs12739979
rs472495
rs41294821
rs7546522
rs6691964
rs146273942
rs11587071
rs55637835
rs17111503
rs530804537
All
−0.8
−0.4
0.0
0.4
MR leave−one−out sensitivity analysis for
'LDL cholesterol || id:ieu−b−110' on 'OLIGOMEN'

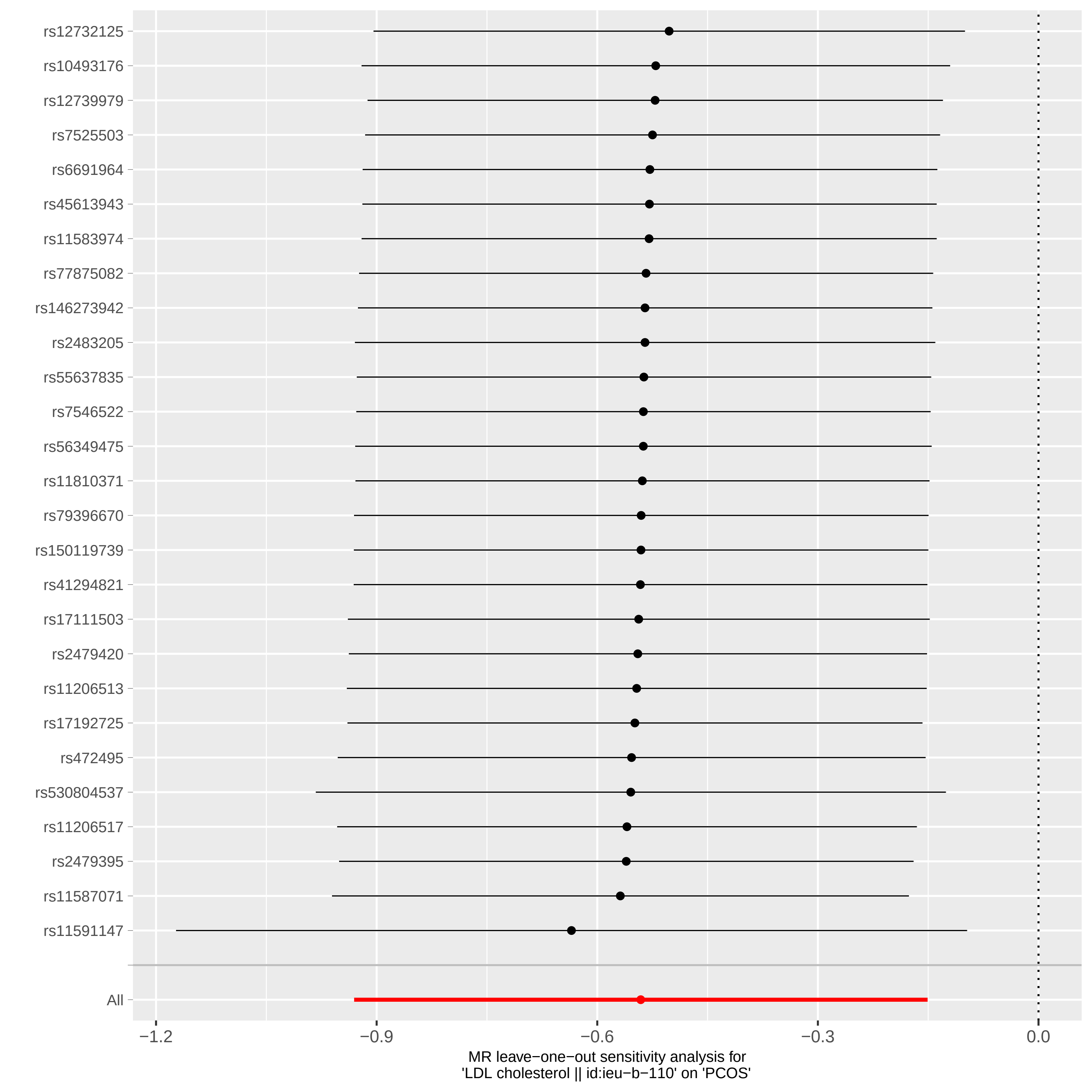
rs12732125
rs10493176
rs12739979
rs7525503
rs6691964
rs45613943
rs11583974
rs77875082
rs146273942
rs2483205
rs55637835
rs7546522
rs56349475
rs11810371
rs79396670
rs150119739
rs41294821
rs17111503
rs2479420
rs11206513
rs17192725
rs472495
rs530804537
rs11206517
rs2479395
rs11587071
rs11591147
All
−1.2
−0.9
−0.6
−0.3
0.0
MR leave−one−out sensitivity analysis for
'LDL cholesterol || id:ieu−b−110' on 'PCOS'

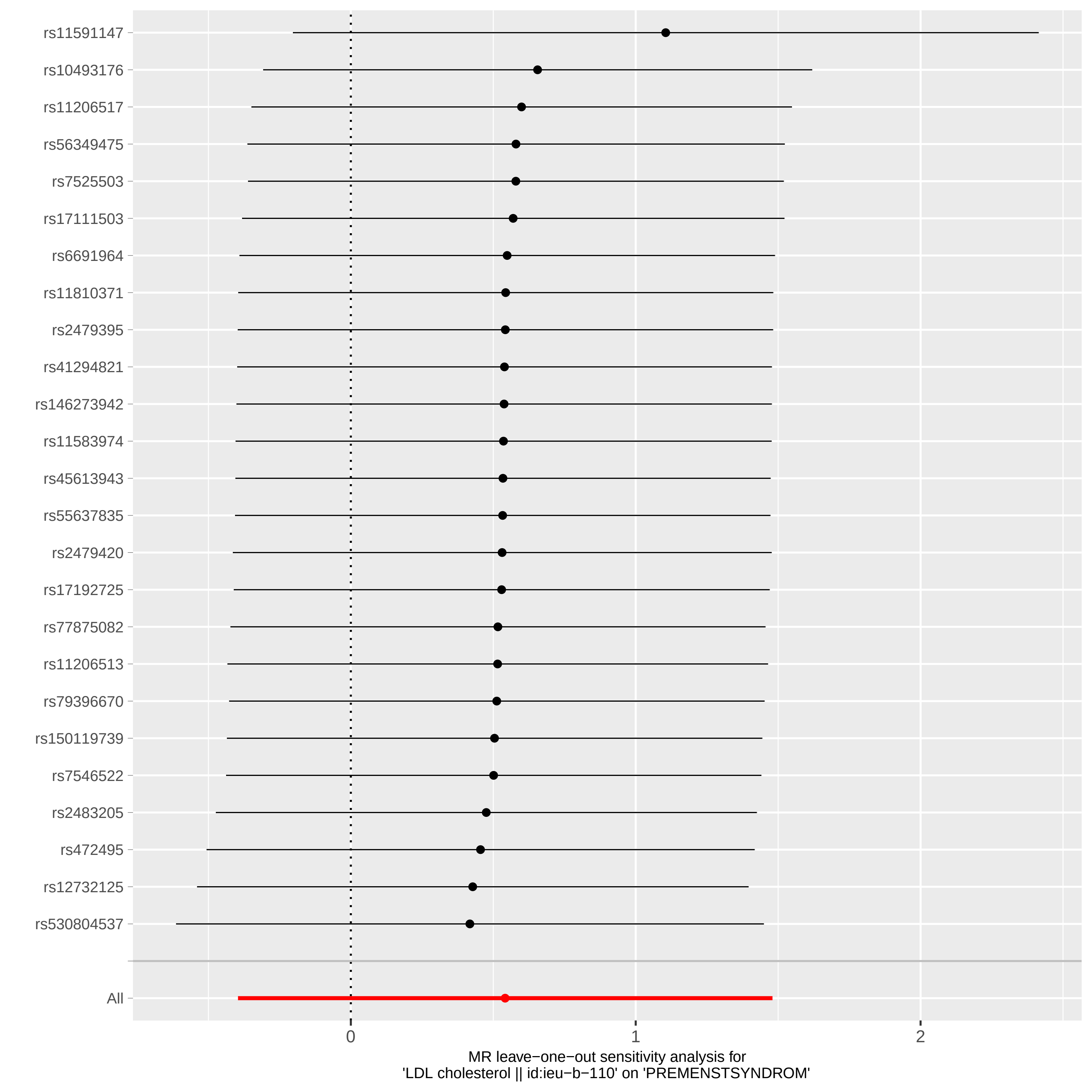
rs11591147
rs10493176
rs11206517
rs56349475
rs7525503
rs17111503
rs6691964
rs11810371
rs2479395
rs41294821
rs146273942
rs11583974
rs45613943
rs55637835
rs2479420
rs17192725
rs77875082
rs11206513
rs79396670
rs150119739
rs7546522
rs2483205
rs472495
rs12732125
rs530804537
All
0
1
2
MR leave−one−out sensitivity analysis for
'LDL cholesterol || id:ieu−b−110' on 'PREMENSTSYNDROM'

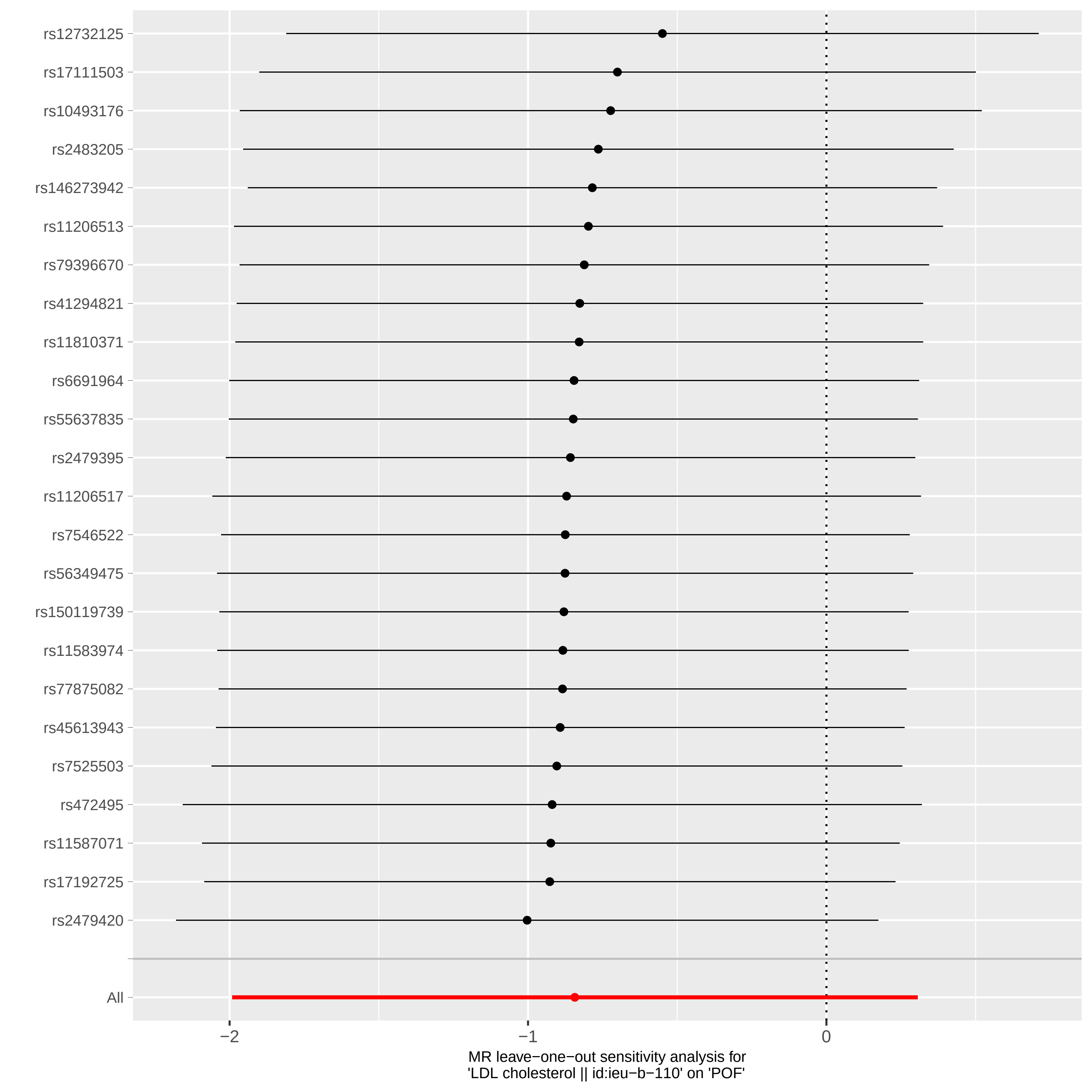
rs12732125
rs17111503
rs10493176
rs2483205
rs146273942
rs11206513
rs79396670
rs41294821
rs11810371
rs6691964
rs55637835
rs2479395
rs11206517
rs7546522
rs56349475
rs150119739
rs11583974
rs77875082
rs45613943
rs7525503
rs472495
rs11587071
rs17192725
rs2479420
All
−2
−1
0
MR leave−one−out sensitivity analysis for
'LDL cholesterol || id:ieu−b−110' on 'POF'
